# Do acute hepatopancreatic necrosis disease-causing PirAB^VP^ toxins aggravate vibriosis?

**DOI:** 10.1080/22221751.2020.1811778

**Published:** 2020-09-02

**Authors:** Phuong Thi Ngoc Tran, Vikash Kumar, Peter Bossier

**Affiliations:** a Lab of Aquaculture & Artemia Reference Center, Department of Animal Sciences and Aquatic Ecology, Faculty of Bioscience Engineering, Ghent University Ghent, Belgium; b ICAR-Central Inland Fisheries Research Institute (CIFRI), Barrackpore, India

**Keywords:** PirAB^VP^ toxin, vibriosis, *Artemia franciscana*, synergistic effect, antagonistic effect

## Abstract

Gram-negative marine bacterium *Vibrio parahaemolyticus* is an important aquatic pathogen and has been demonstrated to be the causative agent of acute hepatopancreatic necrotic disease (AHPND) in shrimp aquaculture. The AHPND-causing *V. parahaemolyticus* strains contain a pVA1 plasmid encoding the binary PirA^VP^ and PirB^VP^ toxins, are the primary virulence factor that mediates AHPND and mortality in shrimp. Since PirAB^VP^ toxins are secreted extracellularly, one can hypothesize that PirAB^VP^ toxins would aggravate vibriosis in the aquatic environment. To address this, *in vivo* and *in vitro* experiments were conducted. Germ-free *Artemia franciscana* were co-challenged with PirAB^VP^ toxins and 10 *Vibrio* spp. The *in vivo* results showed that PirAB^VP^ toxin interact synergistically with MM30 (a quorum sensing AI-2 deficient mutant) and *V. alginolyticus* AQ13-91, aggravating vibriosis. However, co-challenge by PirAB^VP^ toxins and *V. campbellii* LMG21363, *V. parahaemolyticus* CAIM170, *V. proteolyticus* LMG10942, and *V. anguillarum* NB10 worked antagonistically, increasing the survival of *Artemia* larvae. The *in vitro* results showed that the addition of PirAB^VP^ toxins significantly modulated the production of the virulence factors of studied *Vibrio* spp. Yet these *in vitro* results did not help to explain the *in vivo* results. Hence it appears that PirAB^VP^ toxins can aggravate vibriosis. However, the dynamics of interaction is strain dependent.

## Introduction

Shrimp is a highly traded seafood product [1–3]. Intensification of shrimp production has increased the incidence of disease. In the shrimp aquaculture industry, economic losses from disease outbreak have been estimated by the FAO to be over US$9 billion per year, which is approximately 15% of the value of world farmed shellfish production [4,5]. The bacterial disease problem, often vibriosis, has escalated since late 2010, when the industry collapsed in South-Asian countries [6,7]. The *Vibrio harveyi*, *V. alginolyticus*, *V. anguillarum*, *V. splendidus*, *V. salmonicida*, *V. vulnificus*, and *V. parahaemolyticus* strains have been found as the main vibriosis-causative microorganisms [6–9].

Apart from “classical” vibriosis, some *Vibrio* spp. are also responsible for causing acute hepatopancreatic necrosis disease (AHPND), originally known as early mortality syndrome (EMS), having a devastating impact on the shrimp aquaculture industry [10–14]. The shrimp production in AHPND-affected regions has at times dropped considerably (up to 60%) and disease has caused an estimated loss of US $43 billion across Asia (China, Malaysia, Thailand, Vietnam) and in Mexico in last 10 years [15–19]. A *Vibrio* spp. becomes a virulent AHPND-causing strain by acquiring a pVA1 plasmid (63-70 kb) encoding the binary toxin PirA^VP^/PirB^VP^, which consists of two subunits PirA^VP^ and PirB^VP^, homologous to the *Photorhabdus luminescens* insect-related (Pir) toxins PirA/PirB [11]. The secreted binary PirA^VP^ and PirB^VP^ toxins are the primary virulence factor of AHPND-causing bacteria mediating AHPND and mortality in shrimp [19–22]. Notably, some recent studies have reported the presence of PirAB^VP^ carrying pVA1 plasmids in some non-*Vibrio parahaemolyticus* such as *V. campbellii*, *V. owenseii*, and *V. harveyi* [23–26]. That sparks the concern about the influence of AHPND-causing PirAB^VP^ toxin on the virulence of non-AHPND *Vibrio* species, when these toxic proteins are released into the aquatic environment or in the host.

However, the critical problem in studying *in vivo* host-pathogen interaction is the difficulty to either eliminate or extricate the effect of the microbial communities that occur naturally in the system [27]. Additionally, in germ-associated conditions, the microbial communities might influences the physiology of host-associated microbes, thereby making it difficult to understand the host response towards pathogens [28,29]. Consequently, the selection of an appropriate animal model system that permits better delineation of cause and effects in host-pathogen interactions is paramount. The brine shrimp (*Artemia franciscana*), an aquatic invertebrate that can be reared under gnotobiotic conditions (allowing full control over the host-associated microbial communities) are characteristically small, highly osmotolerant, branchiopod crustacean reported from a variety of harsh environment worldwide [27,30–33]. Apart from its unusual life history, relatively low space and cost requirement for culture, rapid generation cycle (cyst to adult in 20–30 days), well-characterized developmental stages and ability to form cyst which can be stored, makes *A. franciscana* an exceptional experimental system to study the host and pathogen relationship [34–37]. In addition, the brine shrimp genome sequence shares high homology with the genomes of shrimps and other crustaceans. Hence, the outcome of *Artemia*-based studies might provide a basis for a better understanding of host-pathogen interactions in commercially important shrimp species [38,39].

In this study, using the highly controlled gnotobiotic brine shrimp model system, it was aimed to investigate whether PirAB^VP^ toxin proteins would aggravate the virulence of 10 *Vibrio* spp., i.e. *V. harveyi* (wild type and 4 quorum sensing deficient mutants, each of them with a certain degree of virulence attenuation relative to the wild type), *V. proteolyticus*, *V. alginolyticus*, *V. campbellii*, *V. anguillarum*, and non-AHPND *V. parahaemolyticus.* Additionally, *in vitro* experiments were also carried out to determine the bacterial cell viability, biofilm formation, swimming motility, haemolytic activity, caseinase activity, and lipase activity in response to PirAB^VP^ toxin. Overall, the results contribute to the understanding of the potential of AHPND toxins to aggravate vibriosis.

## Material and methods

### Bacterial species and growth conditions

Eleven bacterial species including 10 *Vibrio* spp. (*V. harveyi* BB120, BB152, JMH603, MM30, JMH634, *V. alginolyticus* AQ13-91, *V. campbellii* LMG21363, *V. parahaemolyticus* CAIM170, *V. proteolyticus* LMG10942, and *V. anguillarum* NB10) and *Aeromonas hydrophila* LVS3 bacteria were used in the study (Table 1). It is important to mention that the strain *V. harveyi* ATCC strain BAA-1116 (BB120) has been reclassified as *Vibrio campbellii* based on microarray comparative genome hybridization and multilocus sequence analysis [40,41]. The *Vibrio* spp. were used for challenge assay, while *A. hydrophila* LVS3 (autoclaved) served as feed for *Artemia* larvae. The stock culture of bacteria was streaked onto Marine agar (MA) plates (Carl Roth, Belgium) to obtain pure colonies. Afterwards, a single colony was inoculated in a 50 mL Erlenmeyer flask containing 20 mL marine broth (MB) (Carl Roth, Belgium) and incubated overnight at 28°C under constant agitation (120 rpm). The stock culture of each bacterial species was prepared using 30% glycerol and stored at −80°C until further use.

**Table 1.** Bacteria species used in this study.

Bacteria species
*Aeromonas hydrophila* LVS3
*Vibrio harveyi*
BB120 (wild type)
BB152 (Mutation in LuxM: HAI-1 synthase)
JMH603 (Mutation in CqsA: CAI-1 synthase)
MM30 (Mutation in LuxS: AI-2 synthase)
JMH634 (Mutation in LuxM, LuxS and CqsA synthase)
*Vibrio alginolyticus* AQ13-91 (Wild type)
*Vibrio campbellii* LMG21363 (Wild type)
*Vibrio parahaemolyticus* CAIM170 (Wild type)
*Vibrio proteolyticus* LMG10942 (Wild type)
*Vibrio anguillarum* NB10 (Wild type)

### Preparation of recombinant *V. parahaemolyticus* PirA^VP^ and PirB^VP^

The PirA (pET21b PirA^VP^) and PirB plasmid (pET21b PirB^VP^) obtained from Taiwan [10] were transformed into *E. coli* Rosetta (DE3) competent cells and the expression and purification of recombinant PirA^VP^ and PirB^VP^ toxins were done as described by Kumar et al. [21]. Briefly, the expression of C-terminal His6-tagged PirA^VP^ and PirB^VP^ proteins was induced by the addition of 0.25 μM of isopropyl thiogalactoside (IPTG). After expression had proceeded at 16°C for 16 h, the recombinant proteins were purified with the magneHis™ protein purification system (Promega Corporation, USA). The purified recombinant PirA^VP^ and PirB^VP^ toxins were dialyzed in phosphate buffer solution (PBS) (Honeywell, Belgium) and concentrated with Amicon^®^ ultra-15 centrifugal filters (Merck Millipore, USA). The acquired PirA^VP^ and PirB^VP^ toxins were collected and immediately preserved at −80°C for further analysis.

### Detection of *V. parahaemolyticus* PirA^VP^ and PirB^VP^ toxins through SDS-PAGE

After purification, the quality and quantity of the recombinant PirA^VP^ and PirB^VP^ proteins was checked by SDS-PAGE and Bradford method, respectively, as described by Kumar et al. (2019). The proteins were electrophoresed in stain-free 4-20% polyacrylamide gel, with each lane receiving an equivalent volume (10 μL) of toxin. The gels were then stained with Coomassie Biosafe (BioRad Laboratories) and visualized by a ChemiDoc MP imaging system (BioRad, Belgium) for qualitative analysis of recombinant PirA^VP^ and PirB^VP^ toxins. The concentration of protein was determined using the Bradford method [42].

### Gnotobiotic brine shrimp rearing system

The gnotobiotic brine shrimp larvae were produced by hatching brine shrimp cysts axenically (germ-free) following decapsulation and hatching procedures as described by Kumar et al. [43] with slight modifications. Briefly, 30 mg *Artemia franciscana* cysts (INVE Aquaculture, Dendermonde, Belgium) was hydrated in 10 mL distilled water with 2 µm filtered aeration for 1 h. Then, 330 µL NaOH (32%) and 5 mL NaOCl (50%) were added to decapsulate the cysts. After 4 min, the decapsulation was stopped by adding 5 mL autoclaved Na_2_S_2_O_3_ (10 g/L). The decapsulated cysts were washed with filtered autoclaved artificial seawater (FASW) containing 35 g/L instant ocean synthetic sea salt (Aquarium Systems, Sarrebourg, France) to get rid of the decapsulation reagents. The cysts were then suspended in 50 mL falcon tubes containing 30 ml FASW followed by incubating at 28°C on the rotor with constant illumination of about 27 µE/m^2^/sec for 28 h to reach the developmental stage II. After hatching, the axenic condition was checked by spreading 100 µL of hatching water on MA plate and then incubated for 5 days at 28°C [28]. Experiments that started with non-axenic larvae were discarded.

### Brine shrimp challenge assay

To investigate whether it was possible to conduct the challenge test in smaller volume of FASW, a total of three separate *in vivo* assays were performed. At first, the challenge assay was performed in two different containers: glass tubes (10 mL FASW) and Eppendorf tubes (1 mL FASW). The results showed that there was significant difference in the survival of *Artemia* nauplii in the non-challenged (control) group in both glass tubes and Eppendorf tubes. Statistically, the survival rates of *Artemia* nauplii in the control were significantly different from those challenged with *V. harveyi* BB120 (immersion, 10^7^ cells/ml) in two containers after 48 h (Figure S1 A, B). Next, the survival assay was conducted in 96-well plates with 100 µL/well FASW to investigate the survival performance of *Artemia* nauplii in even smaller volume (Figure S1 C). The experiment was performed with three *Artemia* nauplii density, i.e. 1 nauplius/well, 5 and 10 nauplii/well. Average survival rate of *Artemia* nauplii in the control group with a stocking density of 1 nauplius/well was approximately 75% and 25%, in 5 nauplii/well 87% and 35% and 89% and 47% in 10 nauplii/well group after 48 and 60 h post experiment (Figure S1 C). Based on the above results, the 96-well plates containing 10 *Artemia* nauplii in 100 µL FASW were found suitable and used further for challenge experiments in this study. The McFarland standard (BioMerieux, Marcy L’Etoile, France) was used to determine the bacterial density from optical density (OD) assuming 1.2 × 10^9^ cells/ml at OD of 1 [44].

Next, to determine the feeding regime for *Artemia* larvae in 96-well plates during 60 h of the experiment, additional survival assay was performed. Along with previously reported feeding regime, i.e. 10^7^ cells/ml used in 10 and 1 ml FASW challenge assay [21,45], an additional feeding 10^8^ cells/ml was used and fed either once or twice to the *Artemia* larvae. The results indicated that the survival of *Artemia* larvae fed twice was significantly higher than those fed once after 60 h in both feeding doses (Figure S2 A). However, there was no significant difference in *Artemia* survival between two feeding levels, i.e. 10^7^ cells/ml or 10^8^ cells/ml. Afterwards, the *Artemia* larvae were challenged with *V. harveyi* BB120 strain and the survival was recorded after 48 and 60 h post infection. The results showed that feeding doses (10^7^ or 10^8^ cells/ml) did not result in any significant difference in the survival of *Artemia* larvae challenged with *V. harveyi* BB120 strain (Figure S2 B). However, higher survival (%) were recorded in *Artemia* nauplii fed twice at 10^8^ cells/ml as compared with 10^7^ cells/ml feeding regimes, in the challenge assay with *V. harveyi* strains (Figure S3). Therefore, the feeding dose 10^8^ cells/ml, fed twice, was used for further experiment.

After standardization, 96-well plates containing 100 µL FASW, 10 *A. franciscana* larvae per well and feeding twice at 10^8^ LVS3 cells/ml was applied in further challenge assays. In the first experiment, a group of 10 larvae was transferred to 96-well plates and challenged separately with either *V. harveyi* BB120 (wild type), BB152 (HAI-1 deficient mutant), JMH603 (CAI-1 deficient mutant), MM30 (AI-2 deficient mutant), JMH634 (triple QS mutant), *V. alginolyticus* AQ13-91, *V. campbellii* LMG21363, *V. parahaemolyticus* CAIM170, *V. proteolyticus* LMG10942 or *V. anguillarum* NB10 strain (immersion, 10^7^ cells/ml) followed by incubation at 28°C for 60 h. The non-challenge *Artemia* larvae served as control. The survival rate of *Artemia* was recorded every 12 h post challenge. The experiment was carried out in 12 replicates equivalent to 12 wells of the plate. Next, the survival assay was carried out using four experimental groups, i.e. only *Artemia* larvae, *Artemia* larvae + bacterial strain, *Artemia* larvae + PirAB^VP^ toxin (immersion, 5.2 µg/100 µL) and *Artemia* larvae + bacterial strain + PirAB^VP^ toxin (immersion, 5.2 µg/100 µL). In our previous publications, we found that Pir toxins at 5.2 µg/100 µl induce AHPND, damages gastrointestinal tract, causing mortality in brine shrimp larvae [21,46]. Hence, 5.2 µg/100 µL dose was used to further investigate its role on the virulence of non-AHPND *Vibrio* species. The plates were maintained at 28°C for 60 h and every 12 h, the survival rate of *Artemia* was recorded by placing the plates under the microscope. The experiment was carried out in 12 replicates equivalent to 12 wells of the plate.

### Cell viability investigation by flow cytometry

At first, cell viability assay was standardized using different dye and bacterial concentration. It was found that Thiazole Orange and Propium Iodide were detected in FITC (fluorescein isothiocyanate) channel and PC5.5 channel, respectively, in the CytoFLEX flow cytometer system (Beckman Coulter’s Life sciences, France). Hence, the plots of PC5.5-A as the vertical axis and FITC-A as a horizontal axis were used to present the results. The results showed that Thiazole Orange was able to stain both live and dead bacterial cells, while Propium Iodide only stained dead cells (Table S1). Notably, the mixture of both dyes was able to distinguish both live and dead cells in the sample better than those only stained with only one dye (Table S1). Therefore, the mixture of Thiazole Orange and Propium Iodide was used for further cell viability test in this study. Moreover, another experiment was carried out to determine how dead cells of tested bacterial strains locate on the PC5.5 × FITC plot. Subsequently, the logarithm of signal emission from dead cells of the examined strains was at least from 10^3^ to 10^4^ on PC5.5 axis. Particularly, the signal areas of dead cells belonging to *V. harveyi* strains (except for JMH634) and *V. anguillarum* NB10 were gated from 5 × 10^3^ above while to *V. harveyi* JMH634 was at 10^4^ above (Figure S4 A, B). The dead cells of other strains emitted the signal at 10^3^ above in terms of area logarithm (Figure S4 C).

Next, the overnight bacteria cultures adjusted to 10^7^ cells/mL were used for flow cytometry analysis. Bacteria suspension supplemented with 5.2 µg/100 µL PirAB^VP^ toxin protein was used as treatment while the pure suspension served as control. Afterwards, the bacteria suspension was incubated at 28°C for 24 h under constant agitation (6 rpm) followed by centrifugation (2000× *g* at 25°C) for 15 min. Subsequently, the pellets were resuspended in FASW and up to 10^−3^ serial dilution was prepared. Afterwards, 100 µL of acquired suspension was placed into each well of the 96-well plates together with 5 µL Thiazole Orange (17 mM) and 5 µL Propium Iodide (0.15 mM). The mixture of 100 µL of FASW and dyes was used as a negative control. The experiment was conducted in triplicate equivalent to 3 wells of the plate. The plate was then covered by the aluminium foil for 10 min to allow staining reaction to occur. The amount of total count and dead bacterial cells were then determined by CytoFLEX flow cytometer system.

### *In vitro* assay for the biofilm formation, swimming motility, caseinase activity, haemolytic activity, and lipase activity of *Vibrio* spp.

Before performing the *in vitro* assay, the bacterial suspension was diluted to 10^7^ cells/mL using sterile MB and supplemented with PirAB^VP^ toxin at 5.2 µg/100 µL. The bacteria suspension without PirAB^VP^ toxin served as a positive control. Subsequently, a final volume of 500 µL from each group was transferred to Eppendorf tubes and incubated at 28°C for 24 h under constant agitation (6 rpm).

Biofilm formation was quantified as described by Stepanovic et al. [47]. Briefly, the bacterial suspension from two groups described above was adjusted to OD_550_ of 0.1 and 200 µL aliquots was transferred, in triplicate, into the wells of a sterile 96-well plate. Wells receiving only MB served as a negative control. The plate was incubated at 28°C for 24 h under static conditions to allow the biofilm formation. Afterwards, each well was gently washed three times with 300 µL physiological solution (0.9% NaCl) to remove all the non-adherent bacteria. The wells were supplemented with 200 µL of 99% methanol and incubated for 2 h. The methanol was removed from each well and the plate was air-dried overnight. Subsequently, the wells were stained with 150 µL of 0.1% crystal violet for 20 min, rinsed under a gentle flow of tap water and allowed to air dry. Then, wells were supplemented with 150 µL of 95% ethanol to resolubilize the bounded dye from the adherent bacterial cells and absorbance was measured at 570 nm with an infinite 200 Tecan plate reader (Tecan, Switzerland).

Swimming motility was measured as described by Yang and Defoirdt [48]. The bacterial suspension from two groups described above was adjusted to OD_550_ of 0.5 and 2 µL of the sample was spotted at the centre of fresh soft MA (0.2% agar) plates. The pure MB served as negative control. The plates were incubated at 28°C for 24 h. Thereafter, the diameters of the motility halos were measured. Each treatment was conducted in triplicate.

Caseinase activity was measured as described by Natrah et al. [49]. The required volume of medium was divided into two parts, one of which consisted of MB (80 g/L) and agar (30 g/L), and the other half was supplemented with 8% (w/v) skim milk powder. The former was autoclaved at 121°C for 20 min while the latter only needed 5 min of autoclaving. After that, the homogenous mixture of both suspensions was poured into plastic Petri dishes and allowed to air dry. The bacterial suspension from two groups described above was adjusted to OD_550_ of 0.5 and 2 µL of the sample was spotted at the centre of the agar plates. The plates were then incubated at 28°C for 3–4 days. Afterwards, the diameters of the clearing zone and colonies were measured. Each treatment was conducted in triplicate.

Hemolytic activity and lipase activity were measured as described by Natrah et al. [49] on 5% defibrinated sheep blood supplemented with 1% Tween 80 on MA plates. 2 µL of aliquots (OD_550_ of 0.5) were spotted at the centre of the agar plates followed by the incubation at 28°C for 3–4 days. Pure MB was used as negative control. Afterwards, the diameters of activity zones and colonies were observed. Each treatment was conducted in triplicate.

### Statistical analysis

Survival data were arcsine transformed to satisfy normality and homoscedasticity requirements as necessary and then analysed by SPSS software version 24.0. The Independent samples T-test and one-way ANOVA (analysis of variances) followed by Duncan’s multiple range test was used to compare means of survival rate between unchallenged and bacterial challenged shrimp. The interactive effect of PirAB^VP^ toxin and tested bacteria were determined by two-way ANOVA (which allows to subsequently determine if the effect is additive, synergistic, or antagonistic) at 5 different time points. Survival in all treatments had to be higher than 10%, at a certain time point, for an interactive effect to be considered and the interpretation of the results to be reliable. Moreover, the data obtained from *in vitro* tests were analysed by independent samples T-test. The Significance level was set at *p* < 0.05.

## Results

### Virulence of *Vibrio* spp. towards *A. franciscana* larvae

In the first experiment, the virulence of *Vibrio* isolates to the host *Artemia* larvae was investigated by monitoring survival at every 12 h, until 60 h post challenge (Table 2). The results illustrated that the virulence of wild type *V. harveyi* and its derivatives and other studied *Vibrio* isolates were notably different from each other 48 h post challenge. The *Artemia* larvae challenged with *V. proteolyticus* LMG10942, *V. campbellii* LMG21363, *V. parahaemolyticus* CAIM170, *V. harveyi* BB120 (wild type) and BB152 strain (HAI-1 deficient mutant) exhibited significantly high mortality as compared to others *Vibrio* isolates and control 48 h post challenge. Although, the survival of *Artemia* larvae in *V. alginolyticus* AQ13-91, *V. harveyi* MM30 (AI-2 deficient mutant) and JMH603 strain (CAI-1 deficient mutant) group was significantly lower as compared to the control, the *Vibrio*-induced mortality was lower than for other *Vibrio* isolates after 48 h post challenge (Table 2). Interestingly, at 60 h post challenge, a similar trend in the survival of *Artemia* larvae was observed in *V. proteolyticus* LMG10942, *V. campbellii* LMG21363, *V. anguillarum* NB10, *V. parahaemolyticus* CAIM170, *V. harveyi* BB120 (wild type) and BB152 strain (HAI-1 deficient mutant) challenged groups. Moreover, *V. harveyi* JMH634 strain (triple QS mutant) was found to be non-pathogenic and the survival of *Artemia* larvae was similar to the control group. These results indicate that *Vibrio* isolates exhibited a different degree of virulence to *Artemia* larvae. As established before, the virulence of *V. harveyi* and its derivatives were found to be quorum-sensing (QS) dependent [50,51].

**Table 2.** Survival (%) of *Artemia franciscana* at different time points after being challenged with *V. harveyi* wild type and its quorum sensing mutants (mean ± SD, *n* = 12).

Treatments	12 h	24 h	36 h	48 h	60 h
Control	99.2 ± 2.9^a^	99.2 ± 2.9^a^	99.2 ± 2.9^b^	90.8 ± 6.7^e^	62.5 ± 12.2^e^
*V. harveyi* strains					
BB120 (Wild type)	95.8 ± 5.2^a^	95.0 ± 6.8^a^	85.8 ± 9.9^a^	43.3 ± 16.1^c^	7.5 ± 8.7^b^
BB152 (HAI-1^-^)	97.5 ± 6.2^a^	95.0 ± 6.7^a^	88.3 ± 10.3^a^	44.2 ± 10.8^c^	13.3 ± 12.3^bc^
JMH603 (CAI-1^-^)	99.2 ± 2.9^a^	99.2 ± 2.9^a^	96.7 ± 4.9^b^	57.5 ± 8.7^c^	7.5 ± 7.5^b^
MM30 (AI-2^-^)	98.3 ± 3.9^a^	98.3 ± 3.9^a^	97.5 ± 4.5^b^	75.0 ± 7.9^d^	21.7 ± 15.4^c^
JMH634 (triple QS mutant)	97.5 ± 4.5^a^	97.5 ± 4.5^a^	97.5 ± 4.5^b^	91.7 ± 8.4^e^	69.2 ± 9.0^e^
*V. alginolyticus* AQ13-91	99.2 ± 2.9^a^	99.2 ± 2.9^a^	95.8 ± 7.9^b^	60.0 ± 11.3^b^	14.2 ± 9.0^bc^
*V. campbellii* LMG21363	100^a^	99.2 ± 2.9^a^	93.3 ± 9.8^ab^	31.8 ± 15.9^b^	4.2 ± 5.2^b^
*V. parahaemolyticus* CAIM170	100^a^	99.2 ± 2.9^a^	89.2 ± 13.1^a^	38.3 ± 17.5^b^	5.8 ± 5.2^b^
*V. proteolyticus* LMG10942	99.2 ± 2.9^a^	99.2 ± 2.9^a^	92.5 ± 9.7^ab^	11.7 ± 15.3^a^	0.8 ± 2.9^a^
*V. anguillarum* NB10	99.2 ± 2.9^a^	99.2 ± 2.9^a^	97.5 ± 4.5^b^	80.0 ± 6.0^d^	44.2 ± 7.9^d^

Note: The non-challenged group served as control. Significant differences within the column between control and treatment bacterial groups at each sampling point are indicated with different superscript (*p* < 0.05).

### Effect of PirAB^VP^ toxin on virulence factor production and *in vivo* virulence of *V. harveyi* wild type and its isogenic quorum-sensing mutants.

An *in vitro* experiment was conducted aiming to determine the effect of PirAB^VP^ toxin on cellular viability of *V. harveyi* wild type BB120 and mutant strains. The overnight culture of bacteria and PirAB^VP^ toxin mixture was tested for live/dead cells using Thiazole Orange and Propium Iodide staining using flow cytometry. The result showed that addition of PirAB^VP^ toxins significantly increased the total cell count and percentage of dead cells in the suspension of MM30 strain (AI-2 deficient mutant) (Table 3, S2). In contrast, a significant decreased total cell count was recorded in the suspensions of BB152, JMH603, and JMH634 strain supplemented with the toxin. Meanwhile, no significant difference in dead/live cell proportion (dead cells %) was observed in the other *V. harveyi* strains tested (Table 3). The addition of PirAB^VP^ toxins had no effect on BB120 strain cellular viability.

**Table 3.** Average (±SD) cell counts and dead cells percentage of *V. harveyi* wild type BB120 and its derivatives with or without PirAB^VP^ (PirAB^+^ or PirAB^-^), obtained from flow cytometer (*n* = 3).

	Total count (×10^3^ cells/ml)		Dead cells (%)	
	PirAB^-^	PirAB^+^	PirAB^-^	PirAB^+^
BB120 (wild type)	559.3 ± 38.5^a^	500.9 ± 10.3^a^	2.7 ± 0.4^A^	2.4 ± 0.3^A^
BB152 (HAI-1^-^)	792.1 ± 10.0^b^	734.8 ± 5.7^a^	1.5 ± 0.3^A^	1.7 ± 0.2^A^
JMH603 (CAI-1^-^)	650.8 ± 18.5^b^	580.8 ± 12.8^a^	1.5 ± 0.2^A^	1.9 ± 0.1^A^
MM30 (AI-2^-^)	943.2 ± 27.6^a^	1051.0 ± 15.7^b^	1.3 ± 0.1^A^	1.8 ± 0.2^B^
JMH634 (triple QS mutant)	377.9 ± 6.0^b^	144.4 ± 3.7^a^	5.2 ± 0.2^A^	5.0 ± 0.1^A^

Note: Different superscripts indicate significant difference (*p* < 0.05).

The supplementation of PirAB^VP^ toxin significantly enhances the expression of virulence factors, including swimming motility, caseinase activity, and lipase activity of *V. harveyi* BB120 strain (wild type), while the haemolytic activity of bacterium was suppressed in the presence of PirAB^VP^ (Table 4, S3). However, in BB152 (HAI-1 deficient mutant) and MM30 (AI-2 deficient mutant) strains, the addition of PirAB^VP^ toxin significantly enhances the swimming motility and caseinase activity (only in BB152 strain), while biofilm formation, haemolytic activity, and lipase activity was not significantly altered (Table 4, S4, S5). In addition, PirAB^VP^ toxin supplementation increased the biofilm formation and swimming motility of JMH603 (CAI-1 deficient mutant), while increased lipase activity was observed with the JMH634 (triple QS mutant) strain (Table 4, S6, S7). These results indicate that addition of PirAB^VP^ toxin potentially increases the virulence of the studied *V. harveyi* BB120 (wild type), MM30 (AI-2 deficient mutant), BB152 (HAI-1 deficient mutant), JMH603 (CAI-1 deficient mutant) and JMH634 (triple QS mutant) strains, while increased total cell count was only reported from *V. harveyi* MM30 strain.

**Table 4.** The effect of PirAB^VP^ on virulence factors of *V. harveyi* wild type BB120 and its derivatives (*n* = 3).

Category of *in vitro* tests	In the presence of PirAB^VP^				
	BB120 (Wild type)	BB152 (HAI-1^-^)	JMH603 (CAI-1^-^)	MM30 (AI-2^-^)	JMH634 (Triple mutant)
Biofilm formation	NSE	NSE	↑*	NSE	ND
Swimming motility	↑**	↑**	↑***	↑**	ND
Haemolysis activity	↓*	NSE	NSE	NSE	ND
Caseinase activity	↑***	↑**	↓*	↓**	ND
Lipase activity	↑***	NSE	↓*	NSE	↑***

Note: ND = not detected, NSE = no significant effect (*p* > 0.05), ↑: increase, ↓: decrease; *(*p* < 0.05), **(*p* < 0.01), ***(*p* < 0.001).

**Figure 1.**
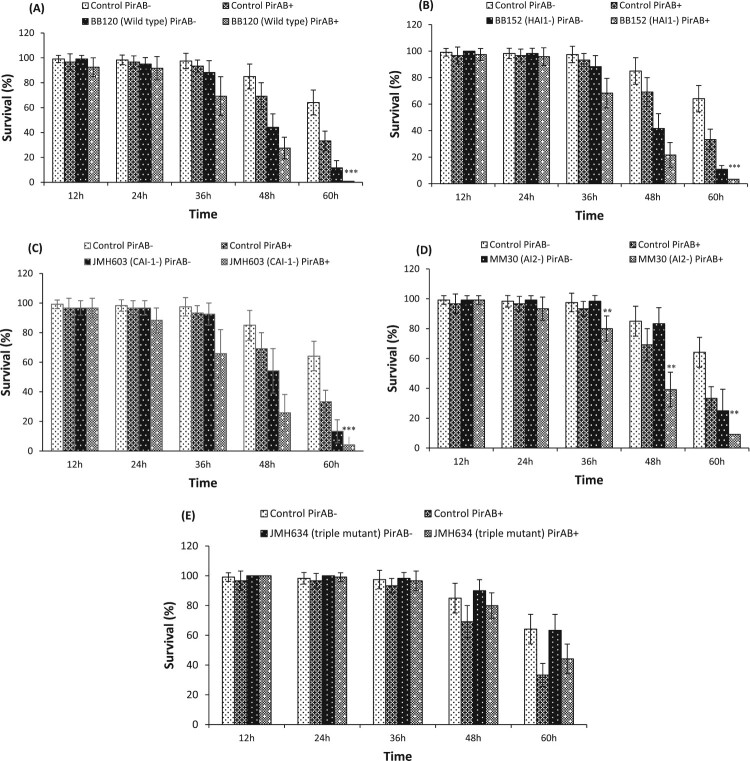
Survival (%) of *Artemia franciscana* at different time points after being challenged with PirAB^VP^ toxin @ 5.2 µg/100 µl and *Vibrio harveyi* strains (A) PirAB^VP^ toxin and *V. harveyi* wild type BB120 strain, (B) PirAB^VP^ toxin and HAI-1 deficient mutant BB152 strain, (C) PirAB^VP^ toxin and CAI-1 deficient mutant JMH603 strain, (D) PirAB^VP^ toxin and AI-2 deficient mutant MM30 strain and (E) PirAB^VP^ toxin and QS triple mutant JMH634 strain. The error bars represent the standard deviation values of 12 replicates. Asterisks on the top of error bars indicate the significant *in vivo* interaction, either synergistic or antagonistic interaction between toxin and bacteria, and based on output interaction plot obtained from two-way ANOVA analysis *(*p* < 0.05), **(*p* < 0.01), ***(*p* < 0.001). The effect of the toxin or the *Vibrio* is not statistically analysed and hence not presented here, only the interactive compound of the 2-way ANOVA is presented. For an interactive statistical effect to be considered of biological relevance, only data at timepoints for which the survival in all treatments was above 10% are considered.

Under the *in vivo* experiment, at first, the quality of the purified PirA^VP^ and PirB^VP^ toxins was analysed through SDS-PAGE. The purified recombinant PirA^VP^ and PirB^VP^ toxins were found at approximately 13 and 50 kDA, respectively, in SDS-PAGE gels (Figure S5). Next, the effect of PirAB^VP^ toxin on the virulence of *V. harveyi* wild type and its derivatives towards *Artemia* was investigated (Figure 1). The results illustrated that PirAB^VP^ toxin challenge in combination with either *V. harveyi* BB120 (wild type), BB152 (HAI-1 deficient mutant), or JMH603 (CAI-1 deficient mutant) display additive effect (no interaction) at 36 and 48 h post challenge and high mortality in *Artemia* larvae was recorded. At 60 h post challenge, the BB120, BB152, or JMH603 strain displayed a statistically significant synergistic interaction with PirAB^VP^ toxin (Figure 1(A–C)). This synergistic effect, towards the end of the challenge period with survival in some treatments becoming very low, might be an inherent feature of survival data with questionable biological meaning. Moreover, significant synergistic interaction of PirAB^VP^ toxin with MM30 (AI-2 deficient mutant) at 36 h (with survival higher than 15% in all treatments) to 60 h post challenge was observed (Figure 1(D)). In contrast, no considerable interaction between PirAB^VP^ toxin and JMH634 (triple QS mutant) strain was detected (Figure 1(E)). These results suggest that PirAB^VP^ toxin can interact with some *Vibrio* strains (more specifically the AI2 mutant, as such mildly virulent towards *Artemia*), increasing its virulence towards *Artemia* larvae while there is no increase in virulence by Pir toxins, when the strain is avirulent (such as the triple QS mutant JMH634).

### Effect of PirAB^VP^ toxin on virulence factor production and *in vivo* virulence of *V. alginolyticus* AQ1391

**Figure 2.**
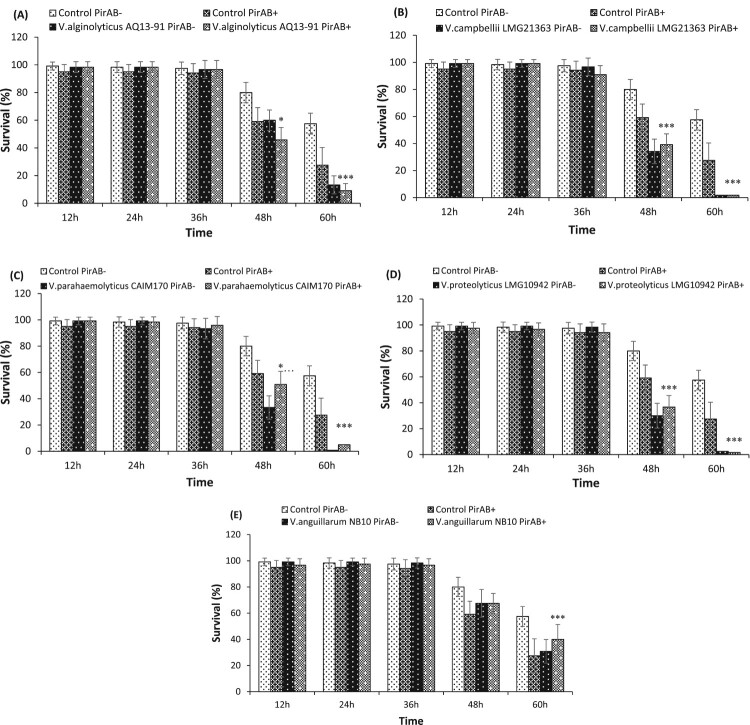
Survival (%) of *A. franciscana* at different time points after being challenged with PirAB^VP^ toxin @ 5.2 µg/100 µl and (A) *V. alginolyticus* AQ13-91, (B) *V. campbellii* LMG21363 (C) *V. parahaemolyticus* CAIM170, (D) *V. proteolyticus* LMG10942 and (E) *V. anguillarum* NB10. The error bars represent the standard deviation values of 12 replicates. Asterisks on the top of error bars indicate the significant *in vivo* interaction, either synergistic or antagonistic interaction between toxin and bacteria, and based on output interaction plot obtained from two-way ANOVA analysis *(*p* < 0.05), **(*p* < 0.01), ***(*p* < 0.001). The effect of the toxin or the *Vibrio* is not statistically analysed and hence not presented here, only the interactive compound of the 2-way ANOVA is presented. For an interactive statistical effect to be considered of biological relevance, only data at timepoints for which the survival in all treatments was above 10% are considered.

Next, the effect of PirAB^VP^ toxin supplementation on *in vivo* virulence of *V. alginolyticus* AQ1391 strain was investigated in *Artemia* larvae. The results showed that in presence of the PirAB^VP^ toxin, *V. alginolyticus* AQ1391 induce significant mortality in *Artemia* larvae (Figure 2(A)). In addition, a significant synergistic interaction between PirAB^VP^ toxin and *V. alginolyticus* AQ1391 was observed at 48 and 60 h post challenge.

The *in vitro* results illustrated that PirAB^VP^ toxin had no significant effect on the proportion of dead cells of *V. alginolyticus* AQ1391 strain as compared to the control (*p* = 0.082490). However, the total cell count in the PirAB^VP^ toxin supplemented group was notably higher than the control (Table 5, S8). The, *V. alginolyticus* AQ1391 did not display biofilm formation, haemolytic, caseinase and lipase activity (Table 6, S9). Taken together, the results showed that the addition of PirAB^VP^ toxin significantly increases the *in vivo* virulence and total cell count of *V. alginolyticus* AQ1391 strain. In contrast, PirAB^VP^ toxin had no effect on dead/live cell proportion and *in vitro* virulence of *V. alginolyticus*.

**Table 5.** Average (±SD) cell counts and dead cells percentage of *V. alginolyticus* AQ1391, *V. campbellii* LMG21363, *V. proteolyticus* LMG10942, *V. parahaemolyticus* CAIM170 and *V. anguillarum* NB10 strains with or without PirAB^VP^ (PirAB^+^ or PirAB^-^), obtained from flow cytometer (*n* = 3).

Bacterial species	Average cell count (×10^3^ cells/ml)		Dead cells (%)	
	PirAB^-^	PirAB^+^	PirAB^-^	PirAB^+^
*V. alginolyticus* AQ13-91	4267.0 ± 67.8 ^a^	5942.7 ± 72.8^b^	2.1 ± 1.2 ^A^	0.5 ± 0.1^A^
*V. campbellii* LMG21363	4339.1 ± 36.7^a^	5639.7 ± 62.4^b^	0.5 ± 0.0 ^B^	0.3 ± 0.0^A^
*V. parahaemolyticus* CAIM170	3383.4 ± 143.2 ^b^	2090.8 ± 68.8 ^a^	2.4 ± 0.5^A^	3.0 ± 0.2^A^
*V. proteolyticus* LMG10942	4027.0 ± 82.7^a^	4894.0 ± 69.1^b^	7.1 ± 0.2 ^B^	4.7 ± 0.2^A^
*V. anguillarum* NB10	1197.4 ± 22.7^a^	999.5 ± 46.8 ^a^	1.4 ± 0.2^A^	1.1 ± 0.0^A^

Note: Different superscripts indicate significant difference (*p* < 0.05).

**Table 6.** The effect of PirAB^VP^ on virulence factors of the studied *Vibrio* spp (*n* = 3).

Category of *in vitro* tests	In the presence of PirAB^VP^				
	*V. alginolyticus* AQ1391	*V. campbellii* LMG21363	*V. parahaemolyticus* CAIM170	*V. proteolyticus* LMG10942	*V. anguillarum* NB10
Biofilm formation	ND	ND	ND	ND	ND
Swimming motility	NSE	NSE	↑*	Overgrew	↓*
Haemolysis activity	ND	↑*	↓*	ND	NSE
Caseinase activity	ND	NSE	NSE	ND	NSE
Lipase activity	ND	ND	ND	ND	ND

Note: ND = not detected, NSE = no significant effect (*p* > 0.05), ↑: increase, ↓: decrease; *(*p* < 0.05), **(*p* < 0.01), ***(*p* < 0.001).

### Effect of PirAB^VP^ toxin on virulence factor production and *in vivo* virulence of *V. campbellii* LMG21363

Interestingly, the addition of PirAB^VP^ toxin significantly decreases the *in vivo* virulence of *V. campbellii* LMG21363 toward *Artemia* larvae. The results showed that after 48 h post PirAB^VP^ toxin and *V. campbellii* LMG21363 exposure, the survival of brine shrimp larvae was significantly higher by approximately 5% as compared to larvae only challenged with bacteria (Figure 2(B)).

The cell viability of *V. campbellii* LMG21363 was affected by the presence of PirAB^VP^ toxin. Results showed that dead cells (%) in LMG21363 strain supplemented with PirAB^VP^ toxin was lower than the non-supplemented suspension (PirAB^-^). In addition, the total cell count of LMG21363 strain with PirAB^VP^ toxin was significantly higher than the control (Table 5, S8). Though, *V. campbellii* LMG21363 did not exhibit biofilm formation and lipase activity, the presence of PirAB^VP^ toxin significantly enhanced the haemolytic activity of LMG21363 strain. Moreover, the addition of PirAB^VP^ toxin had no significant effect on swimming motility and caseinase activity of LMG21363 strain (Table 6, S10). These results indicate that, PirAB^VP^ toxin antagonistically interacts with *V. campbellii* LMG21363 strain and significantly decreases its *in vivo* virulence. In addition, the PirAB^VP^ toxin significantly increase the *in vitro* total cell count and haemolytic activity *V. campbellii* LMG21363, while no effect was observed in swimming motility and caseinase activity.

### Effect of PirAB^VP^ toxin on virulence factor production and *in vivo* virulence of *V. parahaemolyticus* CAIM170

The AHPND-causing PirAB^VP^ toxin significantly decreases the *in vivo* virulence of *V. parahaemolyticus* CAIM170 strain (antagonistic interaction of PirAB^VP^ toxin and CAIM170 strain). The results showed that *Artemia* larvae challenged together with PirAB^VP^ toxin and CAIM170 strain had significantly higher survival at 48 h post exposure as compared with larvae challenged with bacteria only (Figure 2(C)).

The PirAB^VP^ toxin had no significant effect on the cell viability of *V. parahaemolyticus* CAIM170 strain. The total cell count and live/dead cells proportion were found similar in CAIM170 strain with or without PirAB^VP^ toxin addition (Table 5, S8). Though, *V. parahaemolyticus* CAIM170 did not exhibit biofilm formation and lipase activity, the addition of PirAB^VP^ toxin was found to increase the swimming motility of bacterium. However, PirAB^VP^ toxin supplementation significantly decreases the haemolytic activity of CAIM170 strain (Table 6, S11). These results indicate that PirAB^VP^ toxin interacts antagonistically with *V. parahaemolyticus* CAIM170 *in vivo* and decreases its virulence. Moreover, PirAB^VP^ toxin enhances the swimming motility of bacterium, while decreases the haemolytic activity of CAIM170 strain.

### Effect of PirAB^VP^ toxin on virulence factor production and *in vivo* virulence of *V. proteolyticus* LMG10942

Similar to *V. campbellii* LMG21363, the PirAB^VP^ toxin supplementation had antagonistic interaction on *in vivo* virulence of *V. proteolyticus* LMG10942 strain towards *Artemia* larvae. The results showed that, after 48 h exposure with PirAB^VP^ toxin and LMG10942 strain, the survival of *Artemia* larvae significantly increased as compared with larvae challenged with bacteria alone (Figure 2(D)).

Next, the effect of PirAB^VP^ toxin on cell viability of *V. proteolyticus* LMG10942 was investigated. The data obtained from flow cytometry analysis illustrated that the addition of PirAB^VP^ toxin significantly increased the total cell count of LMG10942 strain (Table 5, S8). Moreover, the dead cells (%) in LMG10942 were lower in the presence of toxin as compared to control. Among the studied virulence factors, *V. proteolyticus* LMG10942 did not exhibit haemolytic, caseinase, and lipase activies, while the addition of PirAB^VP^ toxin did not affect the swimming motility of *V. proteolyticus* LMG10942 (Table 6, S12). Together, these results show that PirAB^VP^ toxin interacts antagonistically with *V. proteolyticus* LMG10942 *in vivo* and decreases its virulence. Moreover, PirAB^VP^ toxin had no effect on the *in vitro* virulence of *V. proteolyticus* LMG10942.

### Effect of PirAB^VP^ toxin on virulence factor production and *in vivo* virulence of *V. anguillarum* NB10

The results showed that the addition of PirAB^VP^ toxin had no effect on the virulence of *V. anguillarum* NB10 strain in *Artemia* larvae up to 48 h post challenge. The *Artemia* larvae challenged with *V. anguillarum* NB10 with or without PirAB^VP^ toxin was approximately 68 ± 11% and 68 ± 8%, respectively, after 48 h exposure. However, at 60 h post challenge, the supplementation of PirAB^VP^ toxin significantly decreases the virulence of NB10 strain and the average survival (%) of *Artemia* challenged with the mixture of bacteria and toxin was higher than those challenged with only bacteria (Figure 2(E)) (antagonistic interaction).

The addition of PirAB^VP^ toxin had no effect on the cell viability of *V. anguillarum* NB10 strain. The results showed that NB10 strain supplemented with PirAB^VP^ toxin did not cause significant change in the percentage of the dead cells as compared to the control. Notably, the average total cells count without toxin supplementation was significantly higher than those with toxin (Table 5, S8). Moreover, the addition of PirAB^VP^ toxin on *V. anguillarum* NB10 had no effect on studied biofilm formation, lipase activity, haemolytic activity and caseinase activity. In contrast, the presence of PirAB^VP^ significantly reduced the swimming motility of *V. anguillarum* NB10 (Table 6, S13). Though, PirAB^VP^ toxin interacts antagonistically with *V. anguillarum* NB10 strain and decrease its virulence (*in vivo*), the toxin had no significant effect on the *in vitro* production of virulence factors (except swimming motility) of the NB10 strain.

## Discussion

The Gram-negative *Vibrio* species, ubiquitous in estuarine and coastal waters, are opportunistic aquatic pathogen and responsible for causing AHPND and vibriosis in shrimps [8,20]. For instance, *V. harveyi*, *V. alginolyticus*, *V. anguillarum*, *V. splendidus*, *V. salmonicida*, *V. vulnificus*, and *V. parahaemolyticus* causing conventional vibriosis in shrimp farming [6–9]. While, pVA1 plasmid associated *V. parahaemolyticus*, *V. punensis*, *V. harveyi*, *V. owensii*, and *V. campbelli* are reported to cause AHPND and massive mortality, even up to 100%, in shrimp aquaculture [10–12,21,25,26,52,53]. As AHPND-causing bacteria are releasing extracellular PirAB^VP^ toxins, their presence in the aquatic environment, apart from mediating AHPND and mortality in shrimp (@20 µg toxin/g shrimp) [21,54–56], may modulate the intra and inter-species interaction and influence virulence of non-AHPND *Vibrio* species. To this end, brine shrimp (*Artemia franciscana*), an aquatic invertebrate crustacean and a model for crustacean shrimp [28,29,36,39,57] was used to investigate *in vivo* effect of PirAB^VP^ toxin on the virulence of non-AHPND vibrios during challenge studies. Subsequently, *in vitro* experiments were also carried out to determine the bacterial cell viability, biofilm formation, swimming motility, and the haemolytic, caseinase, and lipase activities in the non-AHPND vibrios in response to PirAB^VP^ toxin exposure.

Quorum sensing (QS) is a bacterial intercommunication system that controls the expression of numerous genes, thereby regulating the activities of large group of cells [58–60]. QS system use small signal molecules called autoinducers (AIs) that controls the bacterial bioluminescence, virulence factor expression, biofilm formation, motility, entry into stationary phase, sporulation, and mating [61,62]. In vibrios, three parallel QS system have been discovered: AI-1, a typical acylated homoserine lactones (AHLs), CAI-1 (*V. cholerae* autoinducer 1), and the AI-2, have been designed for inter-species cell-cell communication [63,64]. Hence, we hypothesized that quorum sensing might regulate the PirAB^VP^ toxin-induced *Vibrio* species virulence and subsequent vibriosis in shrimp. To verify this, *V. harveyi* BB120 (wild type), BB152 (HAI-1 deficient mutant), JMH603 (CAI-1 deficient mutant), MM30 (AI-2 deficient mutant) and JMH634 (triple QS mutant) strains were used, each of them with a certain degree of virulence attenuation relative to the wild type. The results showed that addition of PirAB^VP^ toxin synergistically enhanced *in vivo* virulence of *V. harveyi* MM30 only, while no significant interaction was observed in triple QS mutant JMH634 strain (Figure 1(A–D), Figure 2(A)). Moreover, additive effect was noticed in BB120 and BB152 strains. These observations point in the direction of additive effects for strains that are virulent (wild type or strains in which quorum sensing gene deletions do not affect virulence), synergistic effects for strains with an attenuated (intermediate) virulence (MM30) and no affect for avirulent strains like the triple QS mutant. It is possible that the PirAB^VP^ toxins, known to destroy epithelial cells in the *Artemia* gut [21,22,46,65–69], facilitate the entry of MM30 strain, making epithelial cells a barrier to infection for MM30 cells. Additionally, the PirAB^VP^ toxin supplementation also synergistically increases the *in vivo* virulence of *V. alginolyticus* AQ13-91 strain (wild type), an intermediate virulent strain. In both cases more research will be needed to unravel the mechanistic nature of the observed synergistic effect.

However, supplementation of PirAB^VP^ toxin has significant antagonistic interaction with *in vivo* virulence of *V. campbellii* LMG21363, *V. parahaemolyticus* CAIM170, *V. proteolyticus* LMG10942, and *V. anguillarum* NB10 strain (Figure 2(B–E)). The results showed that, after exposure with PirAB^VP^ toxin and *Vibrio* spp., the survival of *Artemia* larvae significantly increased as compared with larvae challenged with bacteria alone. The cellular and humoral components of the immune system present in the digestive tract plays an important role in preventing the potential binding and invasion of incoming pathogen [70]. There are few reports which suggest that the administration of bacterial toxins could serve as cross-protective antigens and provide protection against microbial infection. For instance, Campa-Cordona et al. [71] reported that recombinant PirA-like toxin, when administered through bath immersion, enhances the immune response of shrimp, providing protection against AHPND-causing *V. parahaemolyticus* [71]. In another study, ToxA^VP^ toxin, when administrated to Pacific red snapper *Lutjanus peru* was reported to positively affect the humoral immune response and upregulate the expression of immune genes that protects the red snapper from *V. parahaemolyticus* infection [72,73]. As, PirAB^VP^ toxin were reported to bind epithelial cells of the digestive tract in brine shrimp larvae, the toxin binding might have induced immunological response and prevented the subsequent attachment and/or entry of pathogenic bacteria. Alternatively, as reported in sea bass larvae (*Dicentrarchus labrax*), the gut epithelial enterocytes containing lysosomes mediates intracellular elimination of pathogenic *V. anguillarum* cells [74,75]. And the putative engulfment of bacterial structures through the shedding of intestinal epithelial cells, as an defence strategy in larval fish, were regularly visualized in the gut lumen at the later stage of bacterial challenge [74]. Hence, the observed antagonistic interaction of PirAB^VP^ toxin with *Vibrio spp*., might be based on either toxin-induced enhanced immune response or on shedding of epithelial cells eliminating the pathogenic bacteria, resulting in decreased *in vivo* virulence of *V. campbellii* LMG21363, *V. parahaemolyticus* CAIM170, *V. proteolyticus* LMG10942, and *V. anguillarum* NB10 strain (Figure 2(B–E)). Yet, such interaction did not happen with all the studied *Vibrio* strains suggesting that the interaction is strain-dependent and might be influenced by the virulence level of bacteria, the mechanistic of which remains to be unravelled.

Moreover, a stochastic pattern of interaction was observed in our *in vitro* experiment. The results showed that addition of PirAB^VP^ toxin significantly increase cell count and dead cell (%) in *V. harveyi* MM30, *V. alginolyticus* AQ13-91, *V. campbellii* LMG21363, and *V. proteolyticus* LMG10942, while decreased value was recorded from *V. harveyi* JMH603, *V. parahaemolyticus* CAIM170, and *V. anguillarum* NB10 strain (Tables 3 and 5). Similar to cell viability observations, the *in vitro* virulence results showed that PirAB^VP^ toxin addition significantly increases the swimming motility (*V. harveyi* BB120, BB152, MM30, JMH603, and *V. parahaemolyticus* CAIM170), caseinase activity (*V. harveyi* BB120 and BB152) and lipase activity (*V. harveyi* BB120 and JMH634). However, decreased haemolysis activity in *V. harveyi* BB120 and *V. parahaemolyticus* CAIM170, caseinase activity *V. harveyi* MM30 and JMH603 were also recorded in the presence of PirAB^VP^ toxin (Tables 4 and 6). Though, these observations do not help to explain the *in vivo* results, the preliminary observations suggest that PirAB^VP^ toxin influences the cell viability and *in vitro* virulence of the studied *Vibrio* spp.

In essence, the results presented here underscore that presence of PirAB^VP^ toxin modulates the virulence of *Vibrio* spp. in both *in vivo* and *in vitro* conditions. Although, it remains to be established how PirAB^VP^ toxin interacts with *Vibrio* spp., to the authors’ knowledge, these findings are the first of its kind, possibly pointing towards new insights in the infection process of *Vibrio* spp. in presence of AHPND-causing PirAB^VP^. Since, in recent years the incidence of AHPND mediated by PirAB^VP^ toxin has increased enormously, further studies that focus on the interaction mechanism of PirAB^VP^ toxin and *Vibrio* spp. in commercial shrimp species (e.g. *Litopenaeus vannamei*, *Penaeus monodon* or *Macrobrachium rosenbergii*) will help to better understand PirAB^VP^ toxins and *Vibrio* spp. mediated infection process.

## Supplementary Material

Supporting_information.docx
