# Supplementary material for: Do acute hepatopancreatic necrosis disease-causing PirABVP toxins aggravate vibriosis?
Source: Emerg Microbes Infect. 2020 Sep 2;9(1):1919–32. doi: 10.1080/22221751.2020.1811778 (PMC8284973; doi:10.1080/22221751.2020.1811778)
Supplement: Supporting_information.docx [file TEMI_A_1811778_SM8462.docx]

**Supplementary information**

**C**

**B**

Figure S1: Survival (%) of *Artemia* larvae challenged with *V. harveyi* BB120 in (A) glass tubes, (B) Eppendorf tubes at 48 h post-challenge and in (C) 96-well plates at 48h and 60h post-challenge. (C) Different *Artemia* densities were used in plates, i.e., 1 *Artemia* per well, 5 *Artemia* per well and 10 *Artemia* per well. The non-challenged group served as control. Different letters on top of error bars indicate significant difference (*p* < 0.05). These error bars indicate the standard deviation values.

**B**

**A**

Figure S2: Survival (%) of *Artemia* larvae after 48h and 60h at different feeding regimes. (A) *Artemia* were fed only in the beginning of experiment (1x) or again after 48h (2x) at 10^7^ LVS3 cells/ml (1.00E+07) or 10^8^ LVS3 cells/ml (1.00E+08). (B) The *Artemia* larvae was fed twice at two different final feeding doses (10^7^ cells/ml and 10^8^ cells/ml) and challenged with *V. harveyi* BB120. The error bars indicate the standard deviation values. Alphabets and asterisks above the bars indicate significant difference between trials *(*P* < 0.05), **(*P* < 0.01).

**A**

**B**

Figure S3: Survival (%) of *Artemia* larvae after 48h and 60h challenged with *V. harveyi* BB120, BB152 (HAI-1 mutant), MM30 (AI-2 mutant), JMH603 (CAI-1 mutant). *Artemia* larvae was fed twice at two different final feeding doses including (A) 10^7^ cells/ml and (B) 10^8^ cells/ml. The non-challenged group served as control. Different letters represent significant difference between trials at different time points (*p*<0.05). The error bars indicate the standard deviation values.

Figure S4: The gated regions of dead cells of (A) *V. harveyi* BB120, (B) *V. harveyi* JMH634 and (C) *V. parahaemolyticus* CAIM170


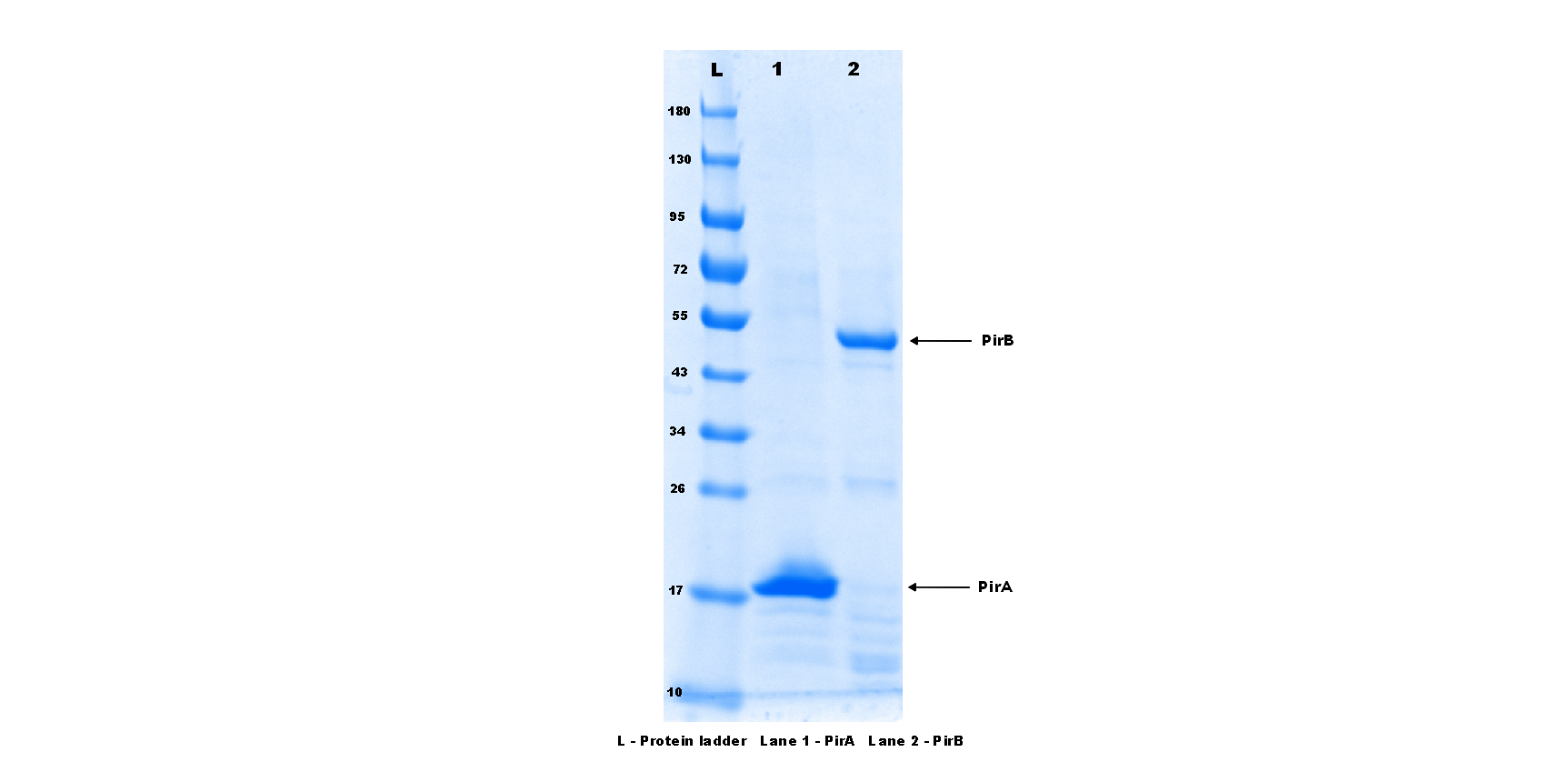


**PirA^VP^**

**PirB^VP^**

**180**

**130**

**95**

**72**

**55**

**43**

**34**

**26**

**17**

**10**

Figure S5. SDS-PAGE analysis of purified *V. parahaemolyticus* PirA^VP^ and PirB^VP^ toxin. Molecular mass standards (M) in kilodaltons (Protein ladder), Lane 1 - PirA^VP^ at 13 kDa, Lane 2 - PirB^VP^ at 50 kDa.

Table S1: Plots of live (in green frame) and dead (in red frame) cells of non-AHPND *V. parahaemolyticus* CAIM170 obtained from flow cytometry staining with only Thiazole Orange, only Propium Iodide or both dyes.

1. **The effect of PirAB^VP^ on cell viability and *in vitro* virulence factors of *Vibrio* sp.**

Table S2: Percentage of live and dead cells of *V. harveyi* wild type and its derivatives without (PirAB^‑^) or with (PirAB^+^) the addition of PirAB^VP^ toxin.

Table S3: The activity zone and growth zone of *V. harveyi* wild type BB120 on plates of different *in vitro* tests together with the corresponding graphs. The error bars of the graphs represent standard deviation. The asterisks above the bars indicate the significant difference (*p*<0.01).

| **Virulence factors** | **Activity zone and growth zone (except Biofilm)** | | **Graphs (if possible)** |
| --- | --- | --- | --- |
|  | **PirAB^-^** | **PirAB^+^** |  |
| Biofilm | Experiment was conducted in 96-well plates, no picture to show | |  |
| Motility | 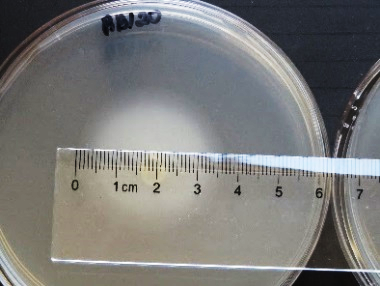 | 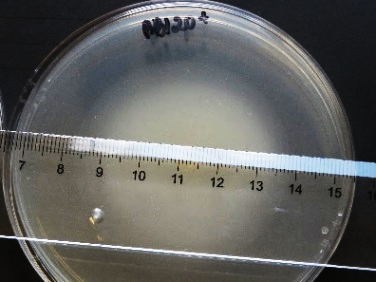 |  |
| Haemolysin | 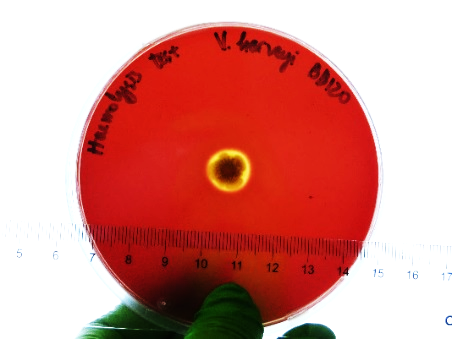 | 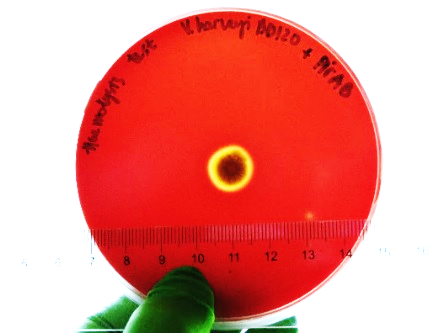 |  |
| Caseinase | 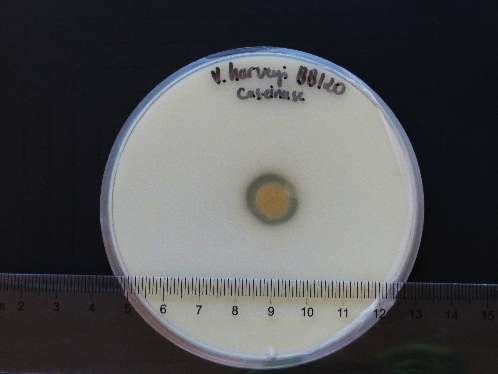 | 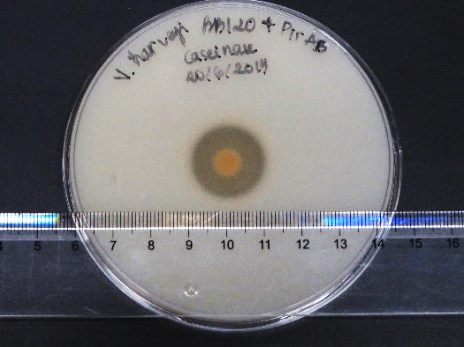 |  |
| Lipase | 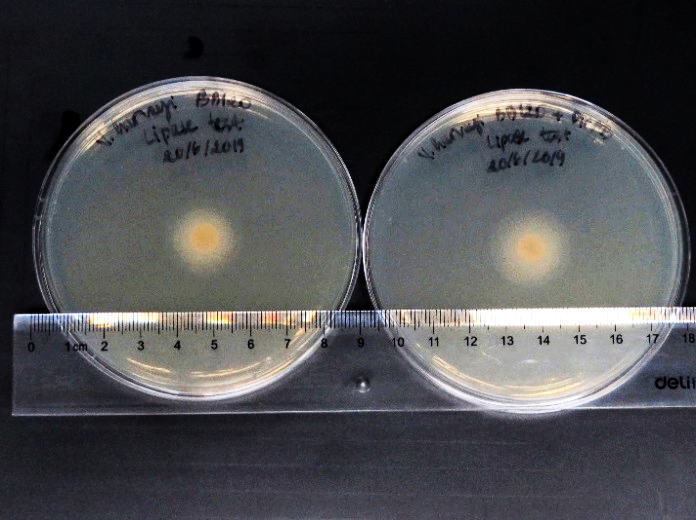 | 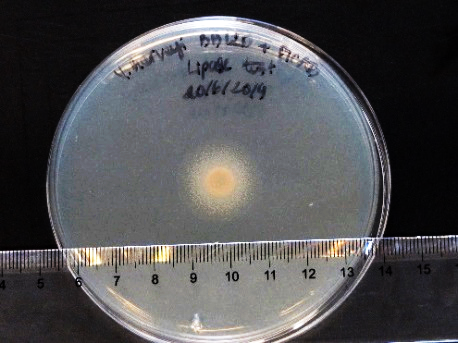 |  |

Table S4: The activity zone and growth zone of *V. harveyi* HAI-1 mutant BB152 of different *in vitro* tests together with the corresponding graphs. The error bars of the graphs represent standard deviation of the mean. The asterisks above the bars indicate the significant difference (*p*<0.01).

| **Virulence factors** | **Activity zone and growth zone on plates** | | **Graphs (if possible)** |
| --- | --- | --- | --- |
|  | **PirAB^-^** | **PirAB^+^** |  |
| Biofilm | Experiment was conducted in 96-well plates, no picture to show | |  |
| Motility | 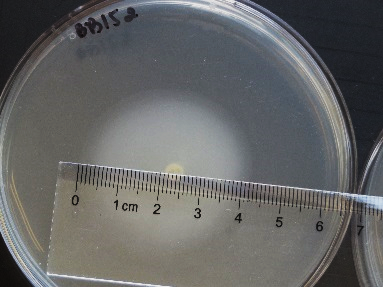 | 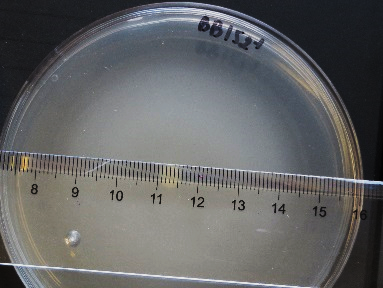 |  |
| Haemolysin | 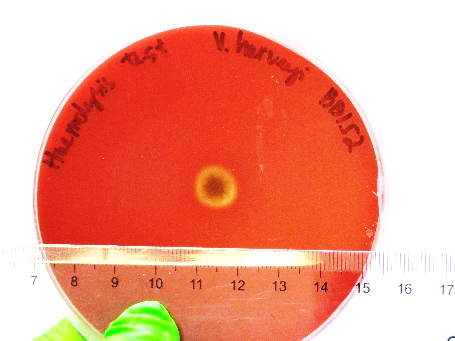 | 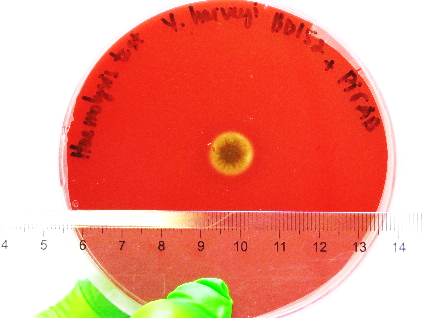 |  |
| Caseinase | 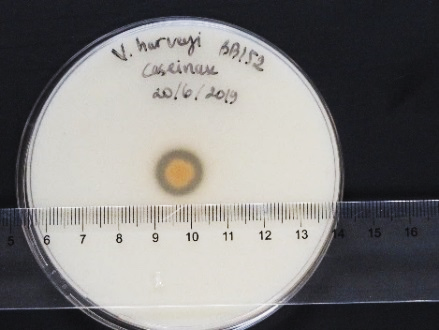 | 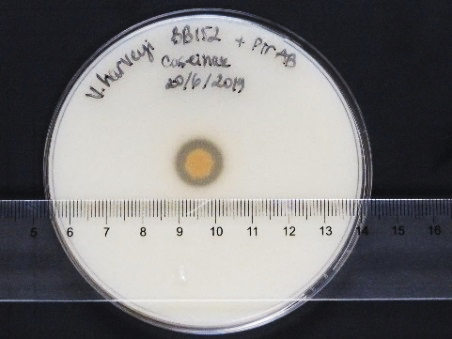 |  |
| Lipase | 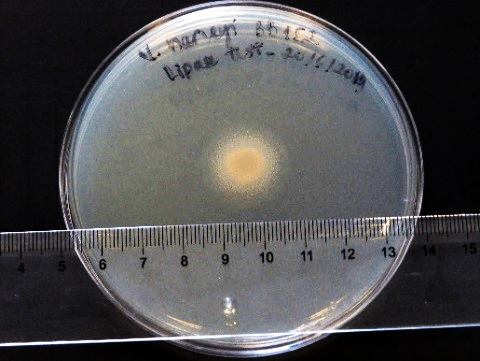 | 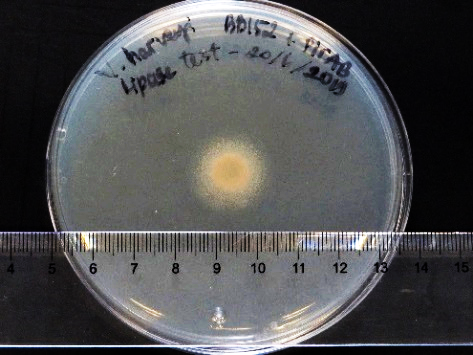 |  |

Table S5: The activity zone and growth zone of *V. harveyi* AI-2 mutant MM30 of different *in vitro* tests together with the corresponding graphs. The error bars of the graphs represent standard deviation of the mean. The asterisks above the bars indicate the significant difference (*p*<0.01).

| **Virulence factors** | **Activity zone and growth zone on plates** | | | | **Graphs (if possible)** | | |
| --- | --- | --- | --- | --- | --- | --- | --- |
|  | **PirAB^-^** | | **PirAB^+^** | |  |  |  |
| Biofilm | | Experiment was conducted in 96-well plates, no picture to show | | | |  |  |
| Motility | | 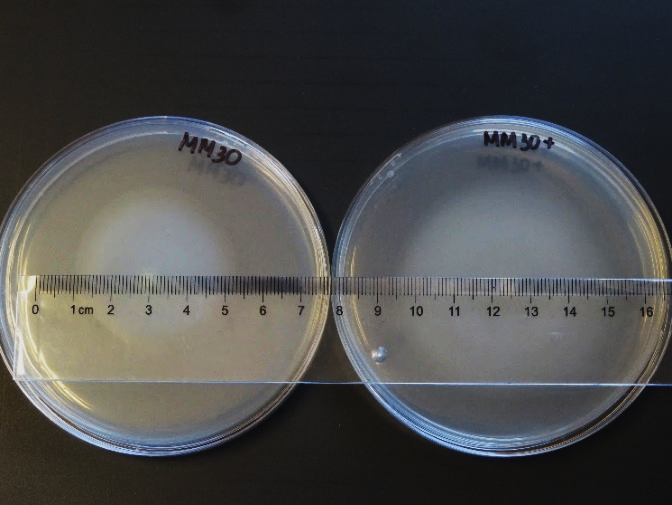 | | 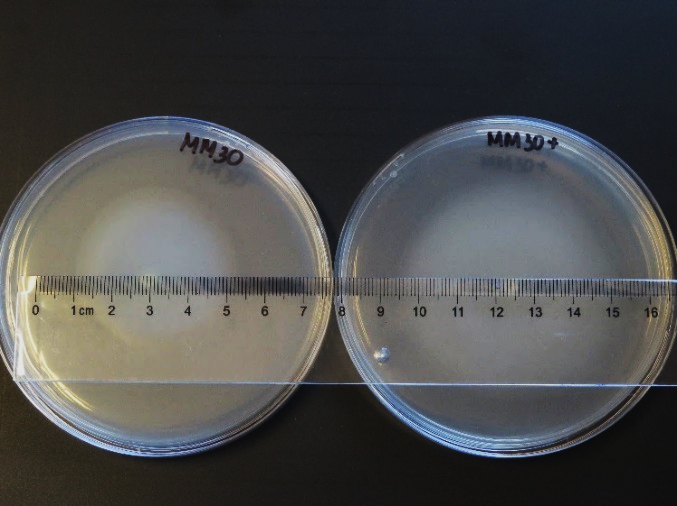 | |  |  |
| Haemolysin | | 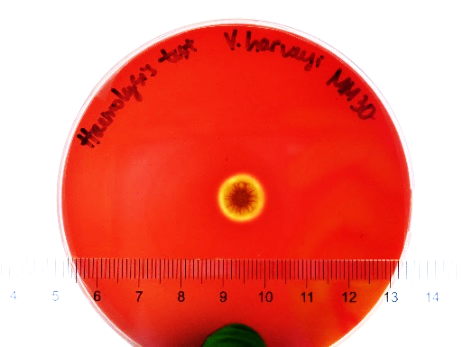 | | 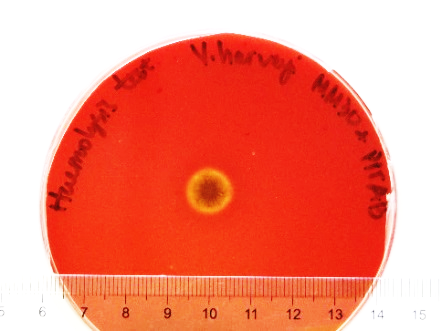 | |  |  |
| Caseinase | | 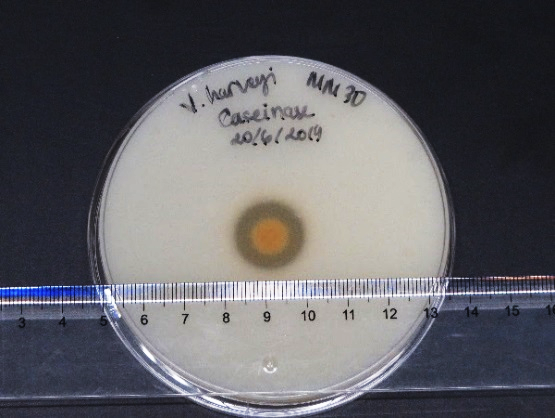 | | 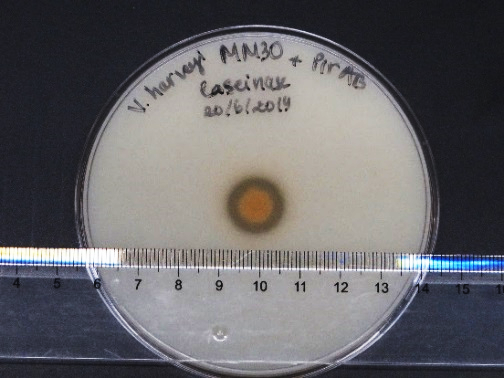 | |  |  |
| Lipase | | 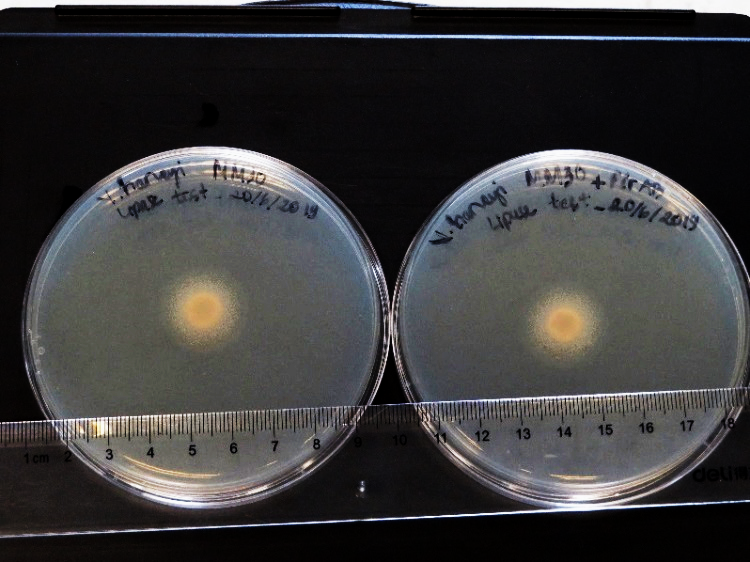 | | 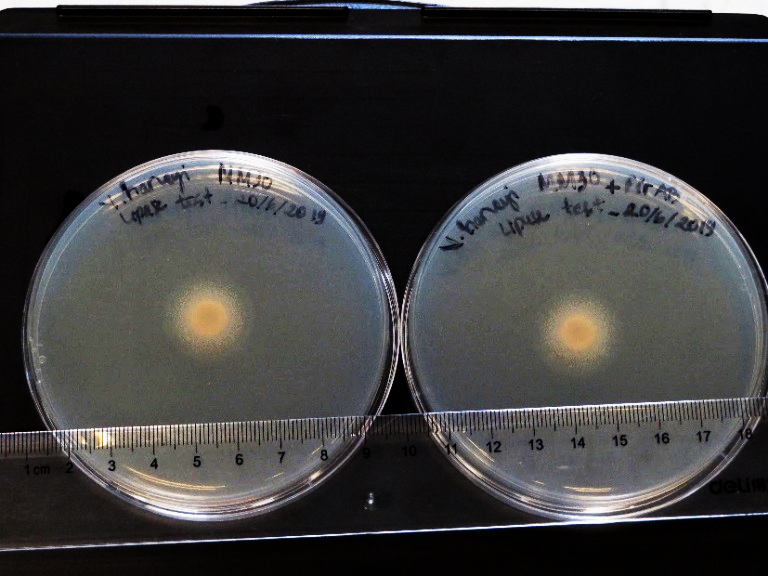 | |  |  |

Table S6: The activity zone and growth zone of *V. harveyi* CAI-1 mutant JMH603 in different *in vitro* tests together with corresponding graphs. The error bars of the graphs represent standard deviation of the mean. The asterisks above the bars indicate the significant difference (*p*<0.01).

| **Virulence factors** | **Activity zone and growth zone on plates** | | | **Graphs (if possible)** |
| --- | --- | --- | --- | --- |
|  | **PirAB^-^** | **PirAB^+^** | |  |
| Biofilm | Experiment was conducted in 96-well plates, no picture to show | | |  |
| Motility | 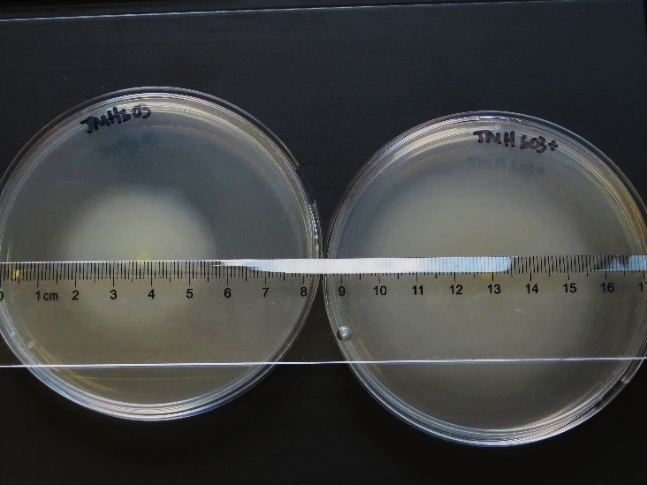 | | 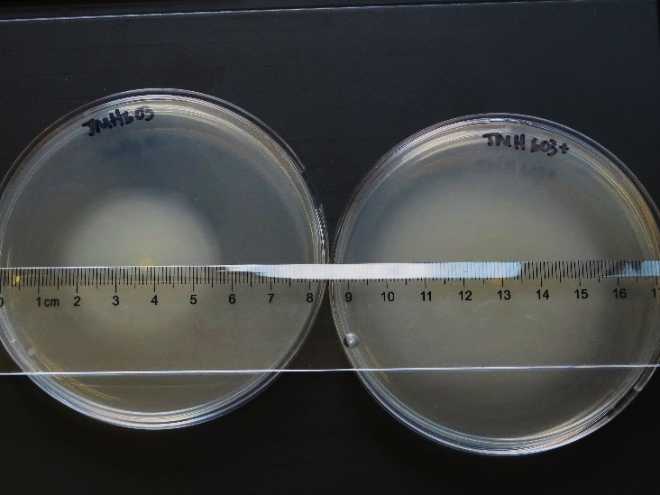 |  |
| Haemolysin | 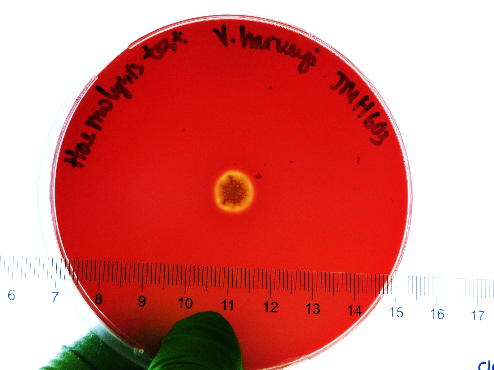 | | 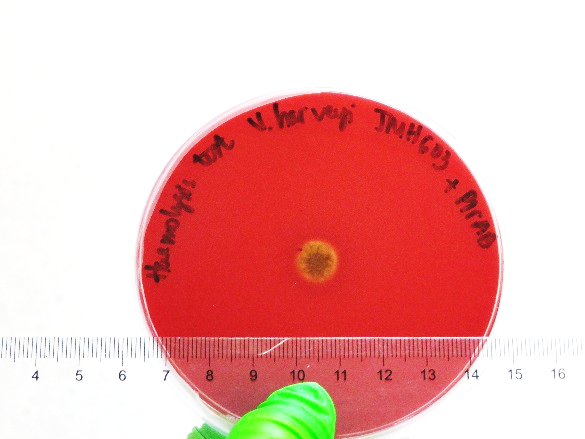 |  |
| Caseinase | 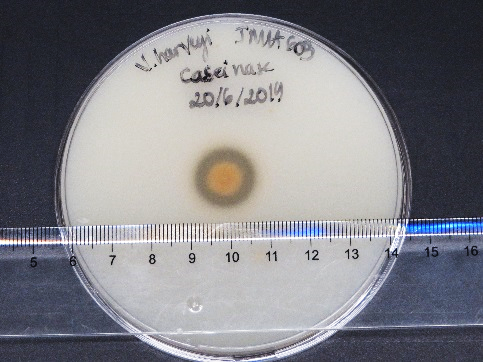 | | 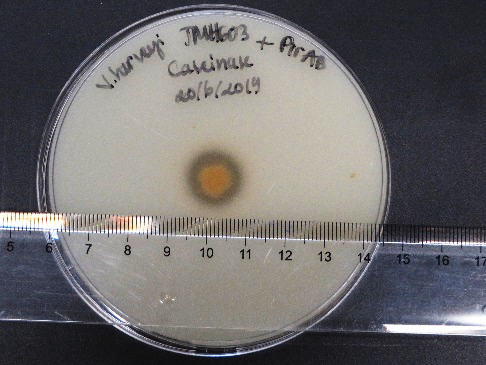 |  |
| Lipase | 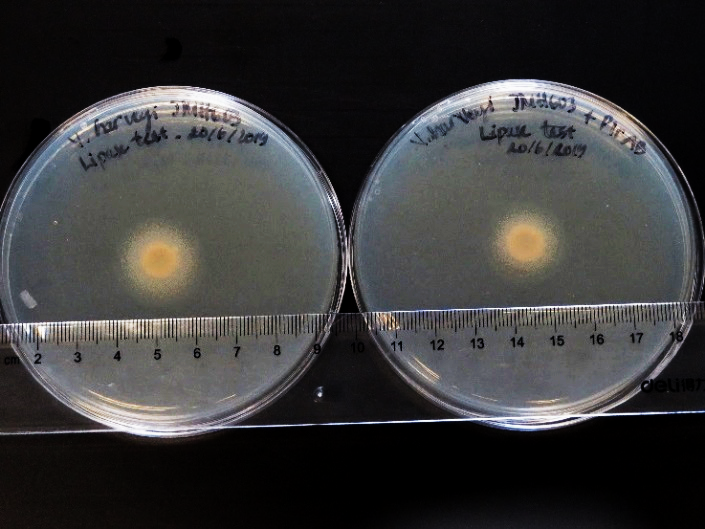 | | 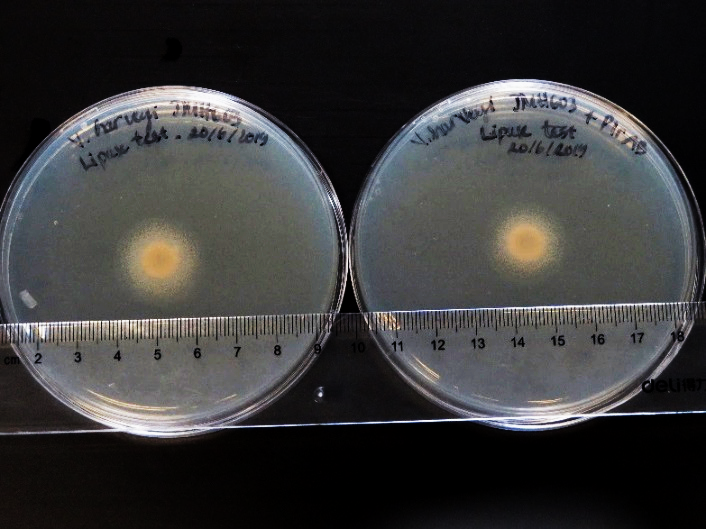 |  |

Table S7: The activity zone and growth zone of *V. harveyi* triple mutant JMH634 in different *in vitro* tests together with corresponding graphs. The error bars of the graphs represent standard deviation of mean. The asterisks above the bars indicate the significant difference (*p*<0.01).

| **Virulence factors** | **Activity zone and growth zone on plates** | | | **Graphs (if possible)** |
| --- | --- | --- | --- | --- |
|  | **PirAB^-^** | **PirAB^+^** | |  |
| Biofilm | Experiment was conducted in 96-well plates, no picture to show | | | No biofilm formed |
| Motility | 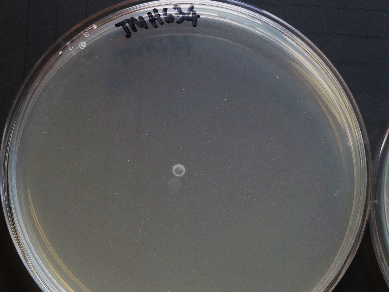 | | 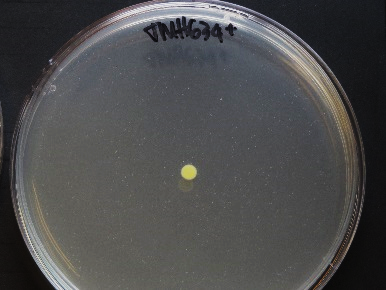 | No activity (no swimming halos) |
| Haemolysin | 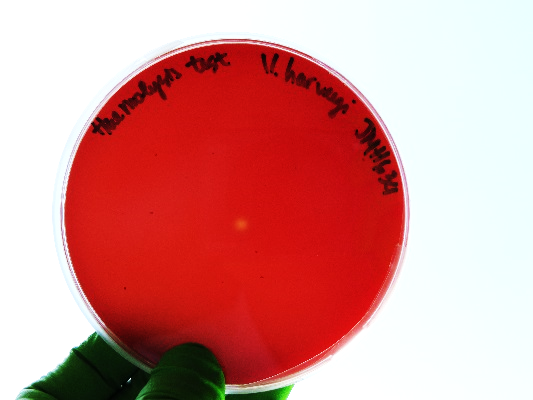 | | 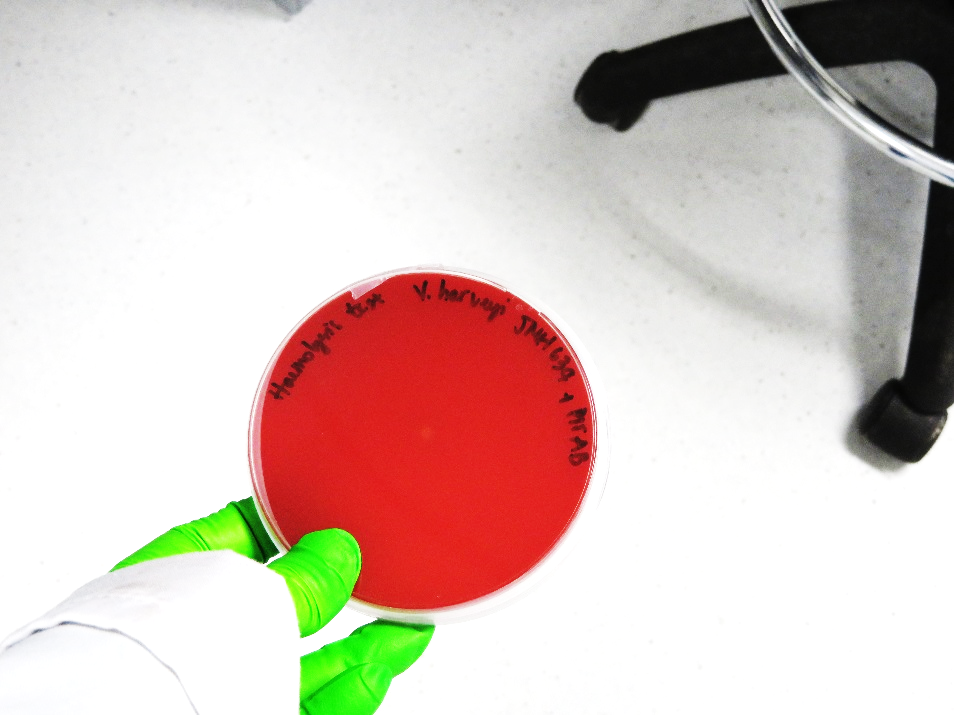 | No activity (no clearing zone) |
| Caseinase | 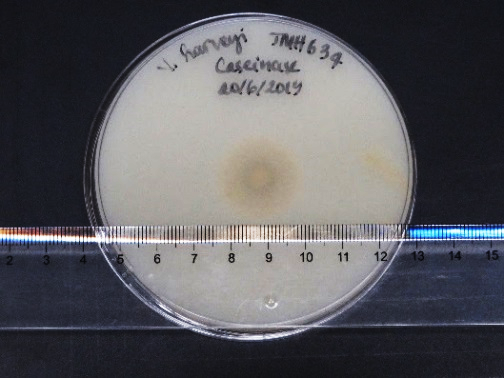 | | 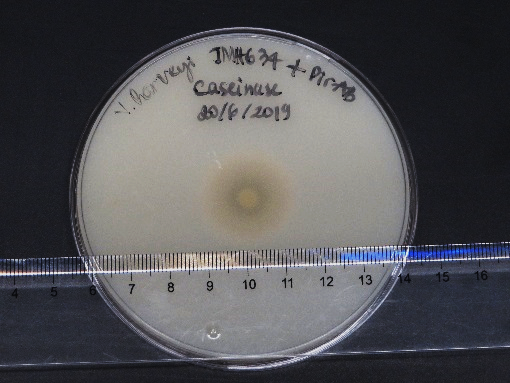 | No activity (no clearing zone) |
| Lipase | 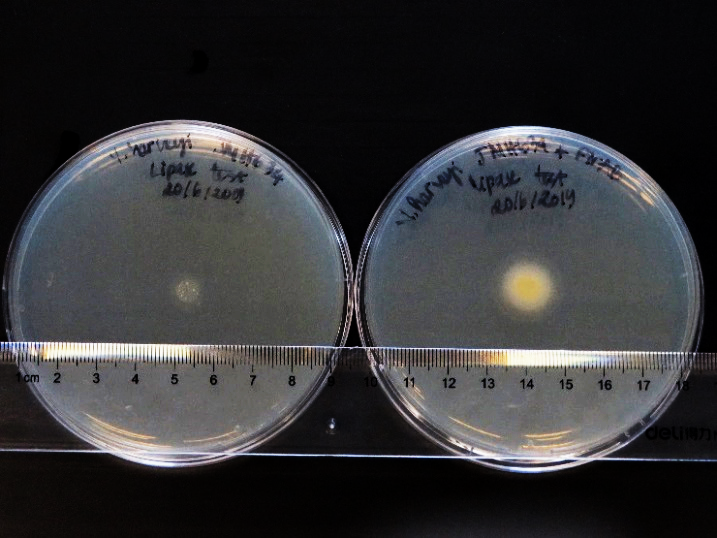 | | 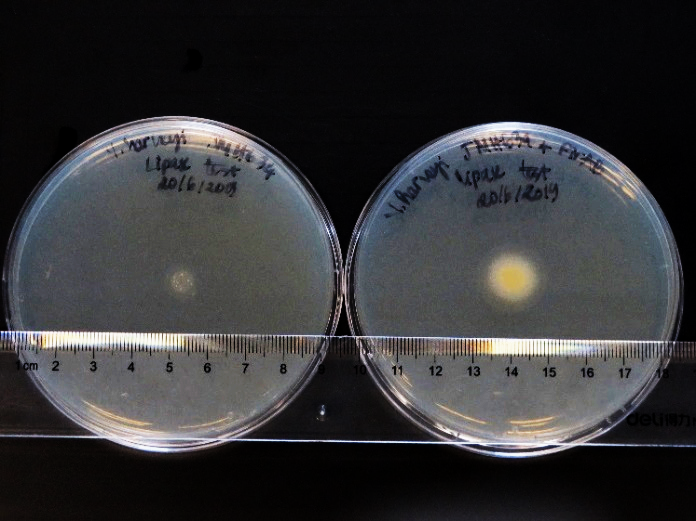 |  |

Table S8: Plots of live and dead cells of *Vibrio* sp. without and with the supplementation of PirAB^VP^ toxins

| **Bacterial strains** | Without toxin(A) or with toxin (B) |
| --- | --- |
| *V. alginolyticus* AQ1391 |  |
| *V. campbellii* LMG21363 |  |
| *V. proteolyticus* LMG10942 |  |
| non-AHPND *V. parahaemolyticus* CAIM170 |  |
| *V. anguillarum* NB10 |  |

Table S9: The activity zone and growth zone of *V. alginolyticus* AQ1391 of different *in vitro* tests together with the corresponding graphs. The error bars of the graphs represent standard deviation of the mean.

| **Virulence factors** | | **Activity zone and growth zone on plates** | | | | | **Graphs (if possible)** | |
| --- | --- | --- | --- | --- | --- | --- | --- | --- |
|  |  | **PirAB^-^** | | **PirAB^+^** | | |  |  |
| Biofilm | | Experiment was conducted in 96-well plates, no picture to show | | | | No biofilm formed | | |
| Motility | **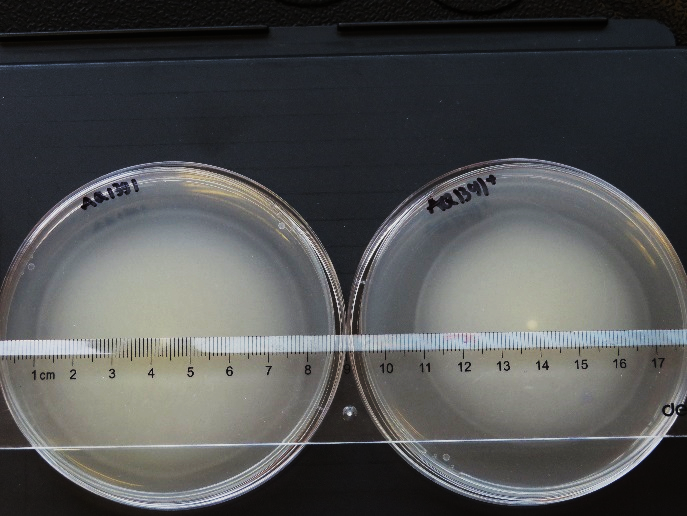** | | **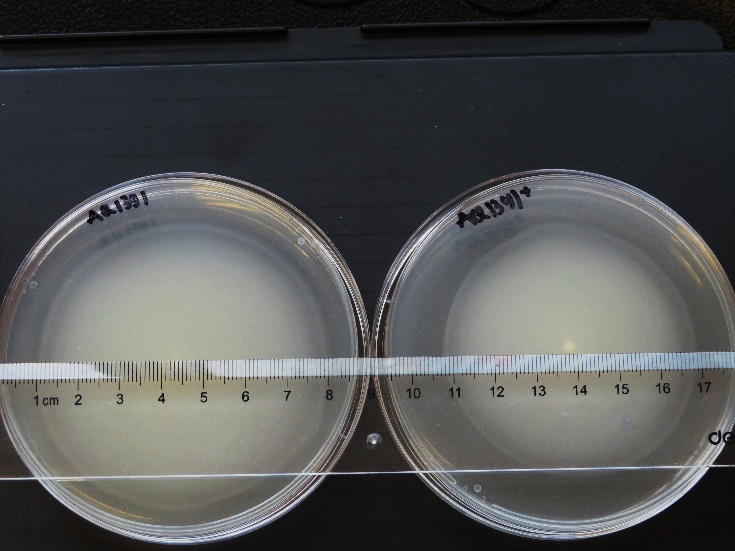** | |  | | |  |
| Haemolysin | 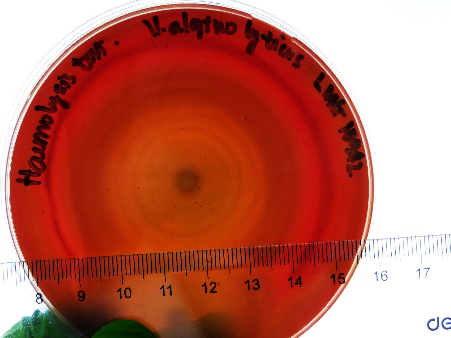 | | 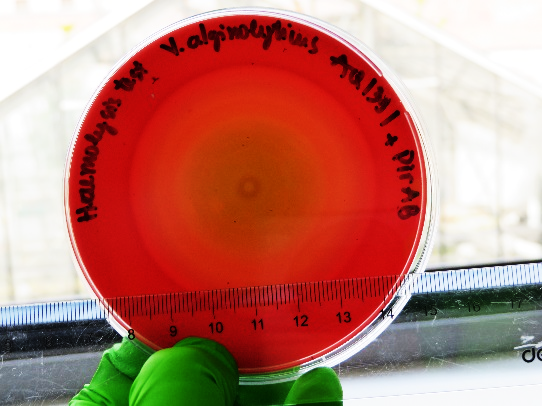 | | No activity (no clearing zone) | | |  |
| Caseinase | **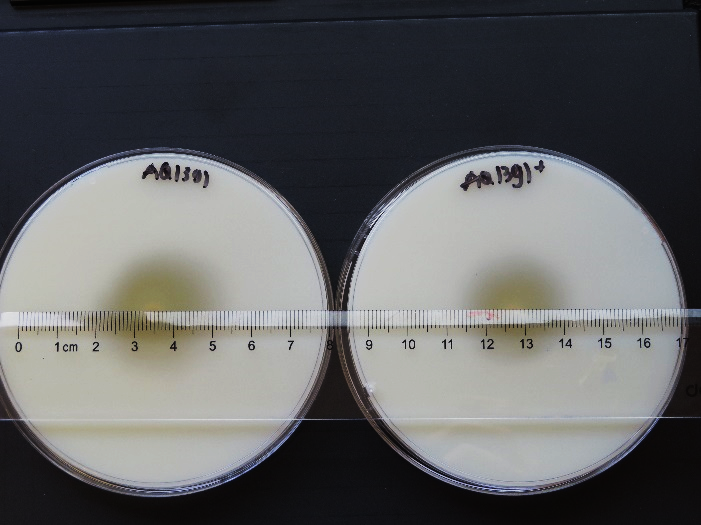** | | **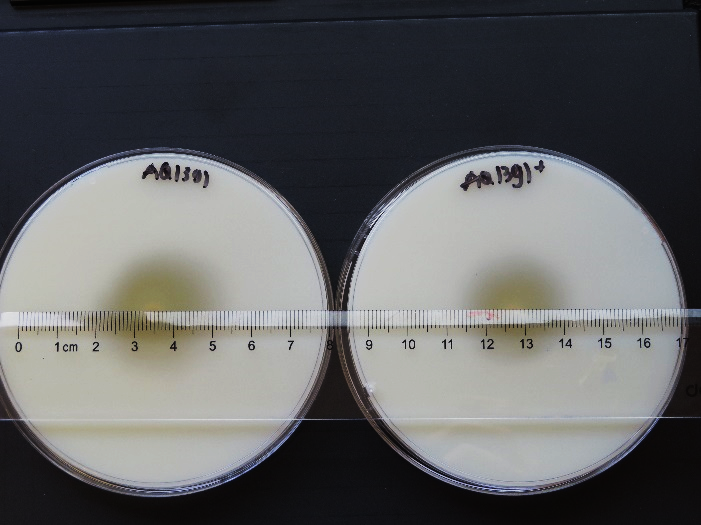** | | No activity (no clearing zone) | | |  |
| Lipase | **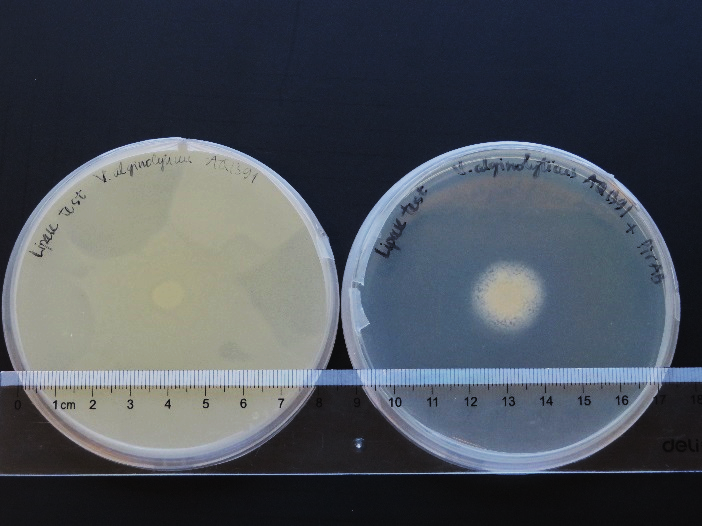** | | **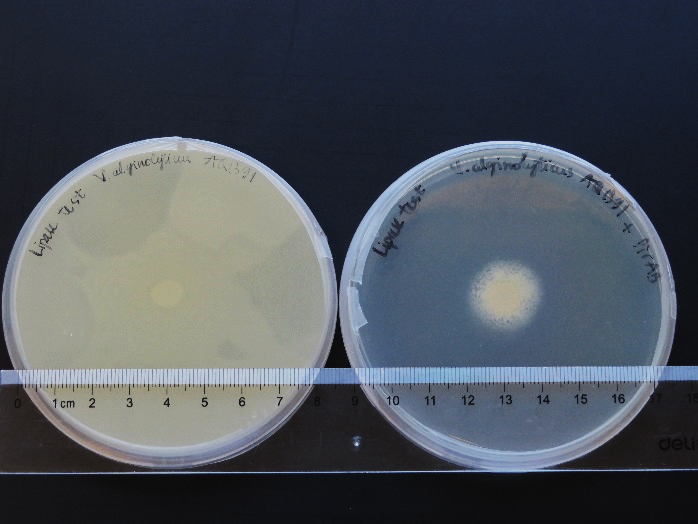** | | No activity (no opalescent zone)  *the addition of PirAB^VP^ seemed to have an inhibiting effect on the growth of bacterial colonies | | |  |

Table S10: The activity zone and growth zone of *V. campbellii* LMG21363 of different *in vitro* tests together with the corresponding graphs. The error bars of the graphs represent standard deviation of the mean. The asterisks above the bars indicate the significant difference (*p*<0.05).

| **Virulence factors** | | **Activity zone and growth zone on plates** | | | | **Graphs (if possible)** | |  |
| --- | --- | --- | --- | --- | --- | --- | --- | --- |
|  |  | **PirAB^-^** | | | **PirAB^+^** |  |  |  |
| Biofilm | | | Experiment was conducted in 96-well plates, no picture to show | | | | No biofilm formed | |
| Motility | 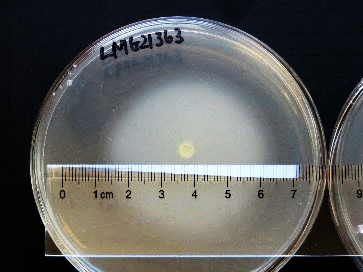 | | | 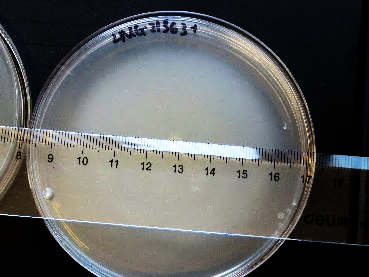 | |  | |  |
| Haemolysin | 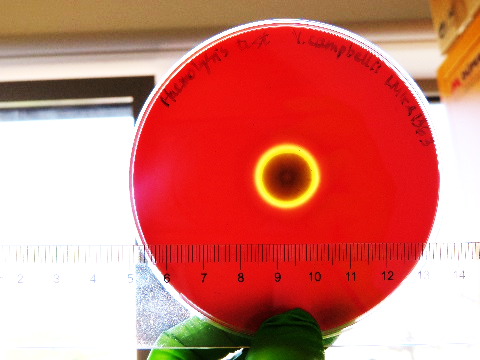 | | | 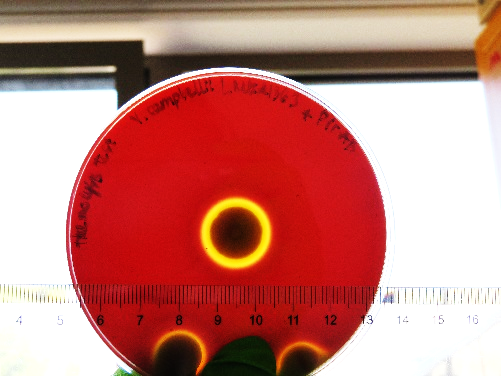 | |  | |  |
| Caseinase | 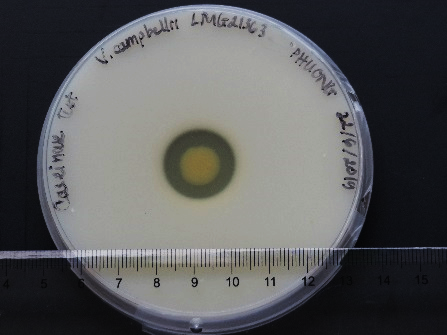 | | | 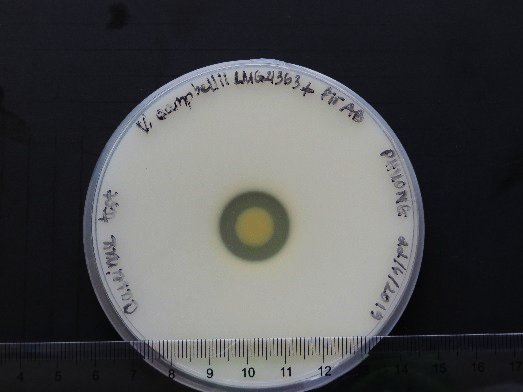 | |  | |  |
| Lipase | 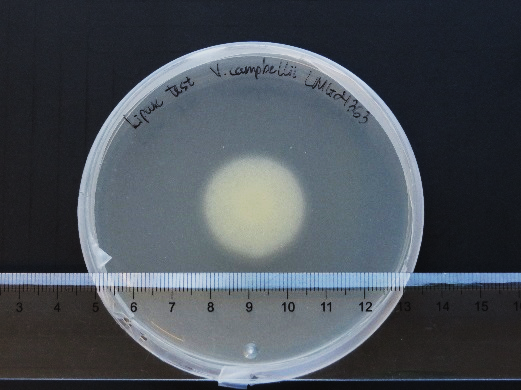 | | | 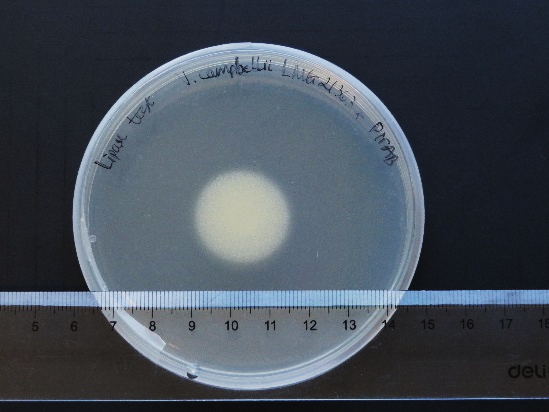 | | No activity (No opalescent zone around the colony) | |  |

Table S11: The activity zone and growth zone of non-AHPND *V. parahaemolyticus* CAIM170 of different *in vitro* tests together with the corresponding graphs. The error bars of the graphs represent standard deviation of the mean. The asterisks above the bars indicate the significant difference (*p*<0.05).

| **Virulence factors** | **Activity zone and growth zone on plates** | | | **Graphs (if possible)** | |
| --- | --- | --- | --- | --- | --- |
|  | **PirAB^-^** | | **PirAB^+^** |  |  |
| Biofilm | Experiment was conducted in 96-well plates, no picture to show | | | | No biofilm formed |
| Motility | **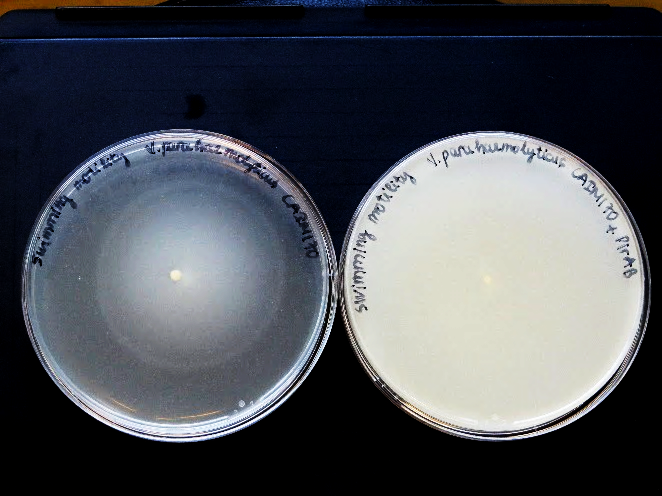** | **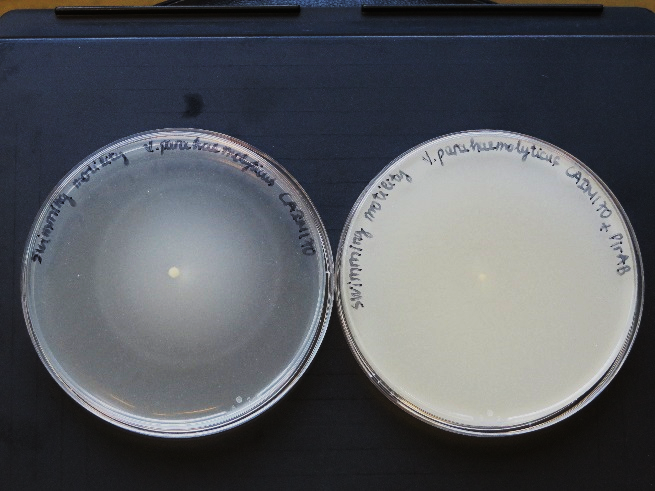** | |  | |
| Haemolysin | 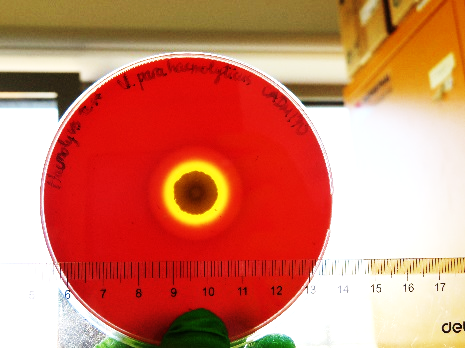 | 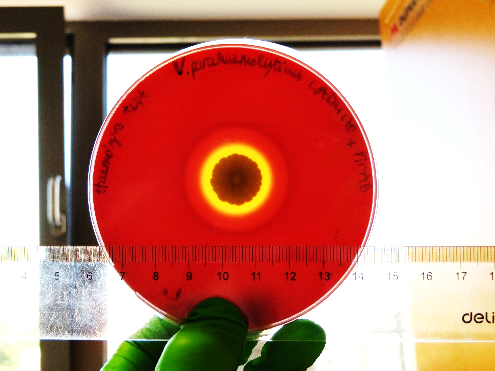 | |  | |
| Caseinase | **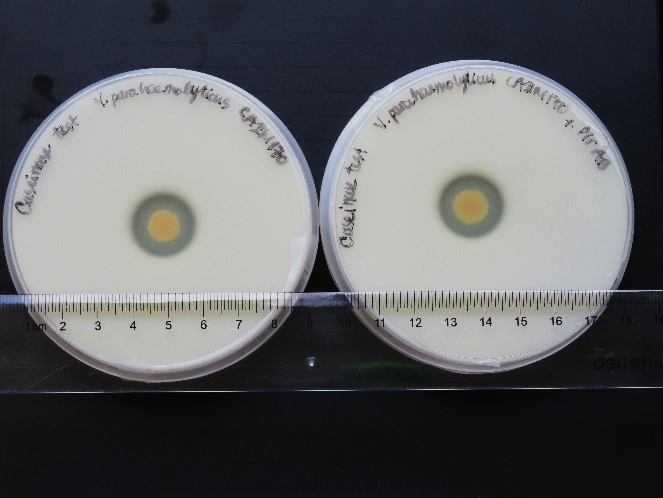** | **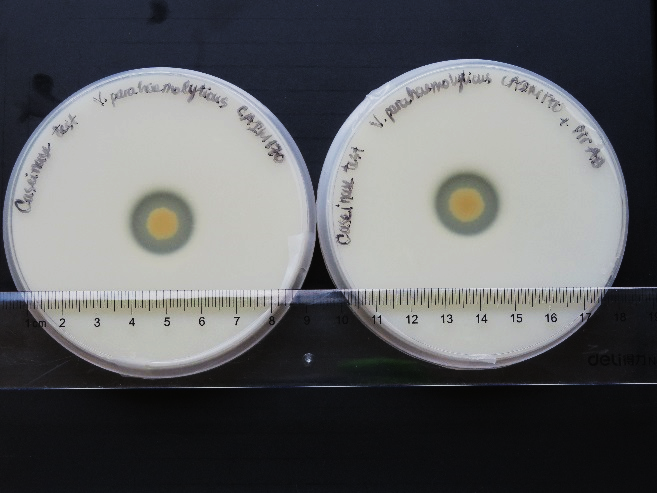** | |  | |
| Lipase | **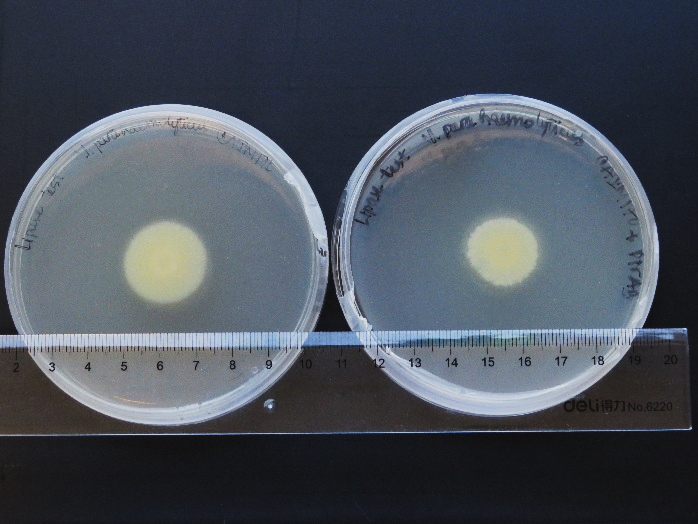** | **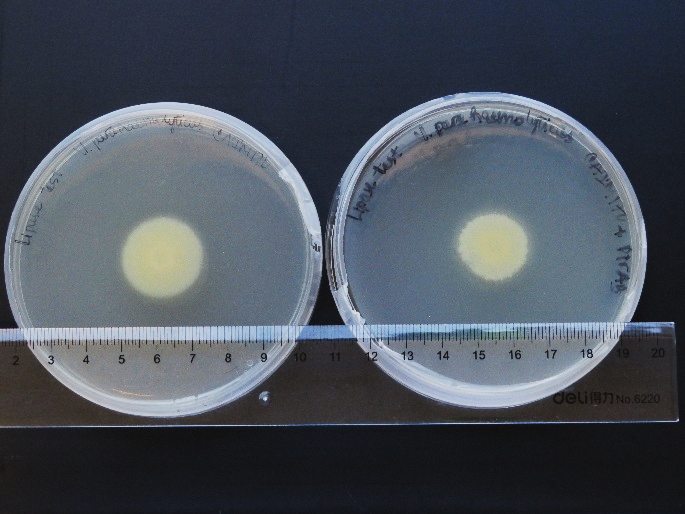** | | No activity (No opalescent zone around the colony) | |

Table S12: The activity zone and growth zone of *V. proteolyticus* LMG10942 of different *in vitro* tests together with the corresponding graphs.

| **Virulence factors** | **Activity zone and growth zone on plates** | | | | | | **Remarks** |
| --- | --- | --- | --- | --- | --- | --- | --- |
|  | **PirAB^-^** | | | | **PirAB^+^** | |  |
| Biofilm | | | Experiment was conducted in 96-well plates, no picture to show | | | | No biofilm formed |
| Motility | | 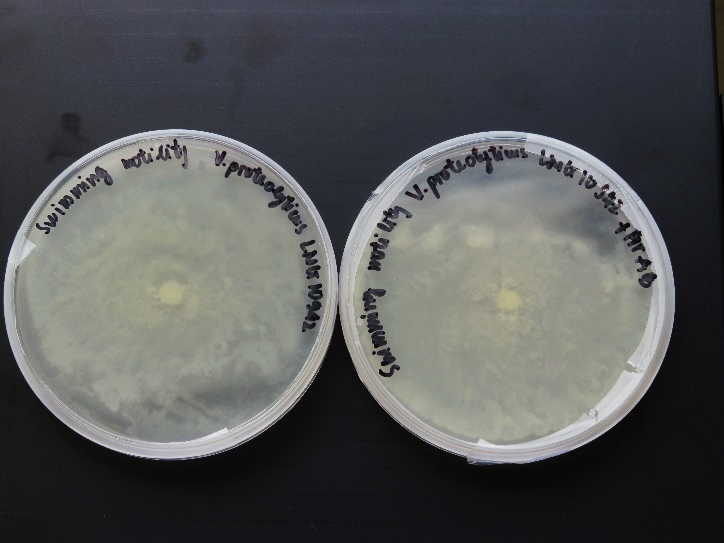 | | 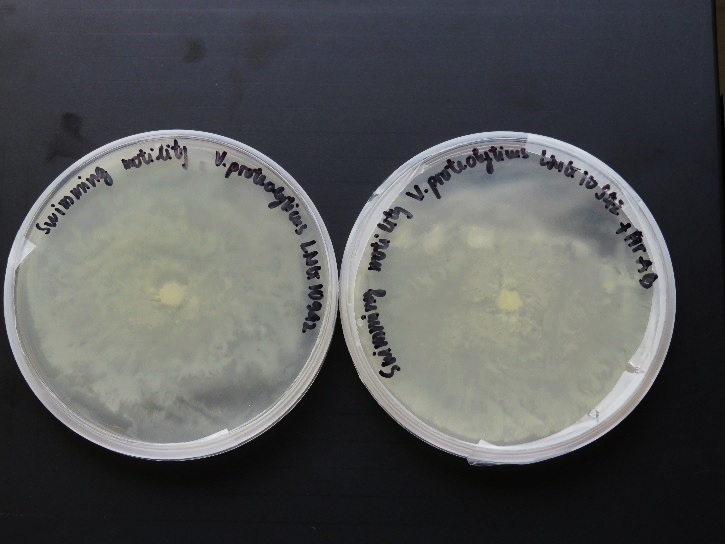 | | *Unable to compare because the colonies reached the edge of plates after 24h of incubation | |
| Haemolysin | | 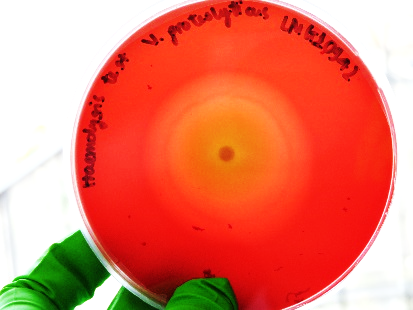 | | 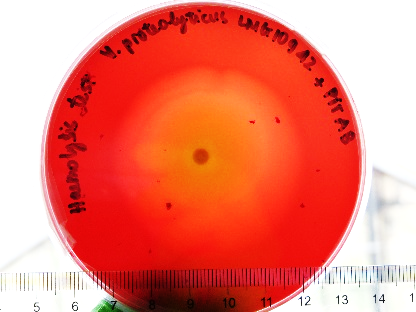 | | No activity (no clearing zone) | |
| Caseinase | | 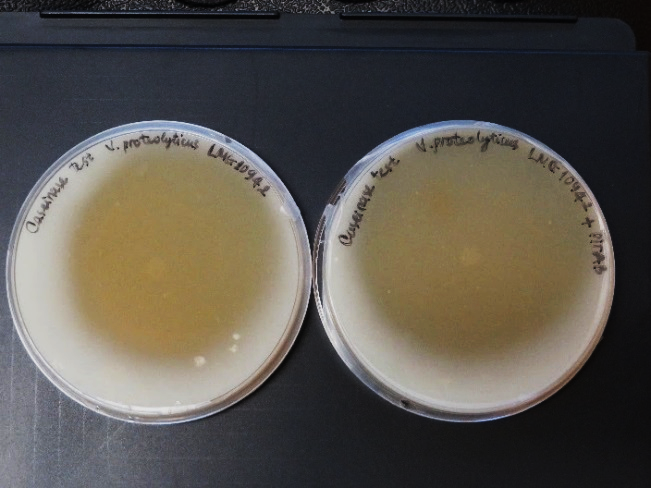 | | 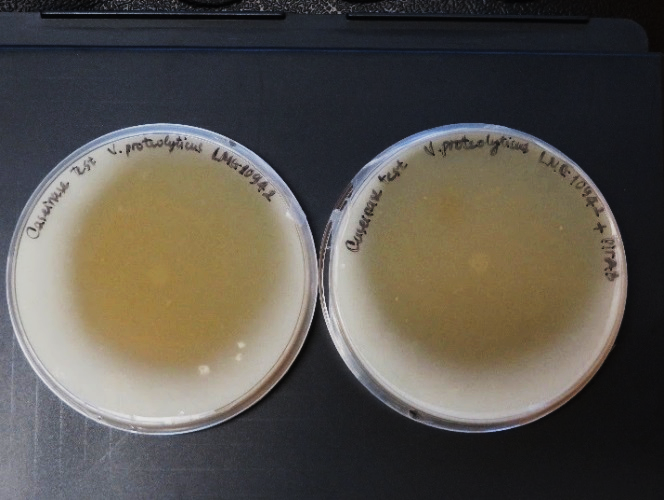 | | No activity (no clearing zone) | |
| Lipase | | 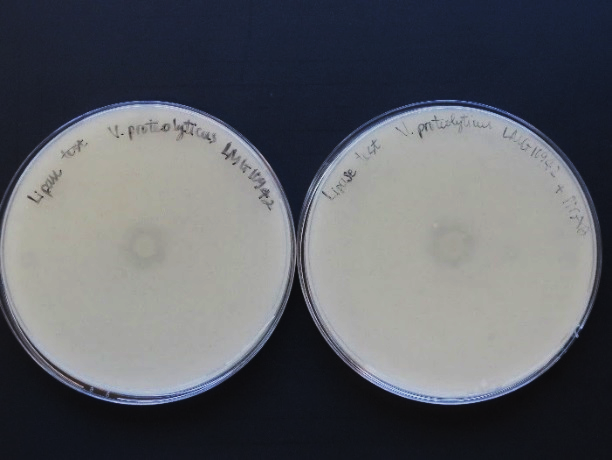 | | 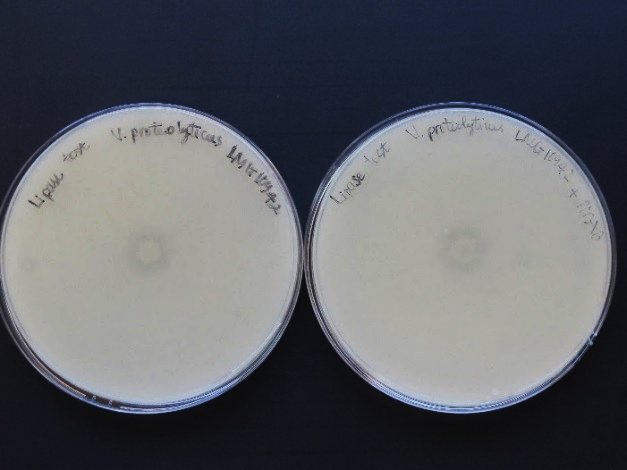 | | No activity (no opalescent zone)  *bacteria overgrew | |

Table S13: The activity zone and growth zone of *V. anguillarum* NB10 of different *in vitro* tests together with the corresponding graphs. The error bars of the graphs represent standard deviation of the mean. The asterisks above the bars indicate the significant difference (*p*<0.05).

| **Virulence factors** | **Activity zone and growth zone on plates** | | | | | | **Graphs (if possible)** | |
| --- | --- | --- | --- | --- | --- | --- | --- | --- |
|  | **PirAB^-^** | | | | **PirAB^+^** | |  |  |
| Biofilm | | | Experiment was conducted in 96-well plates, no picture to show | | | No biofilm formed | |  |
| Motility | | 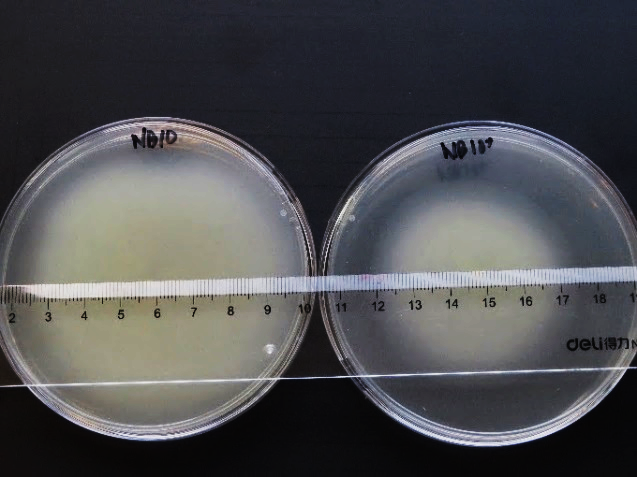 | | 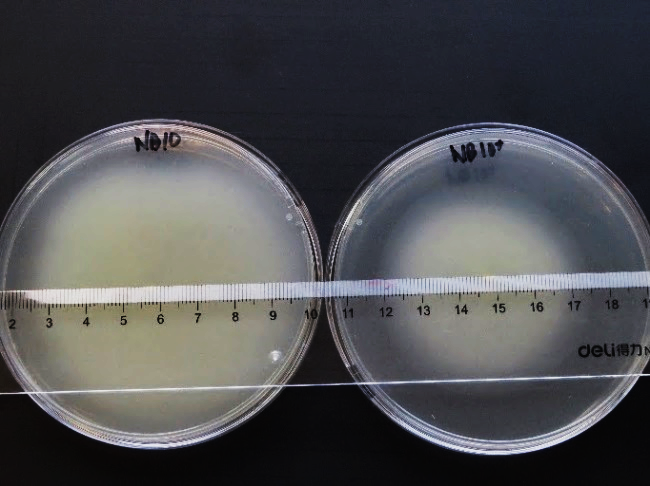 | | |  | |
| Haemolysin | | 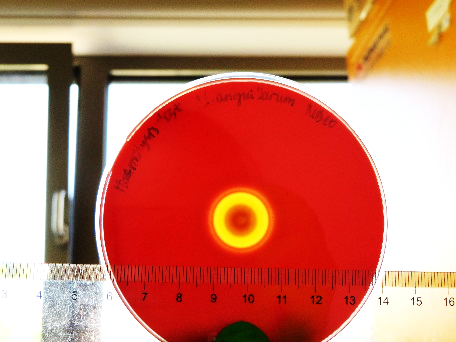 | | 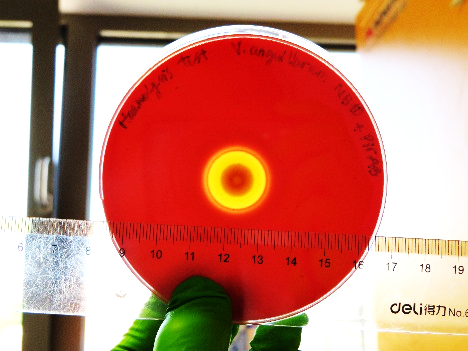 | | |  | |
| Caseinase | | **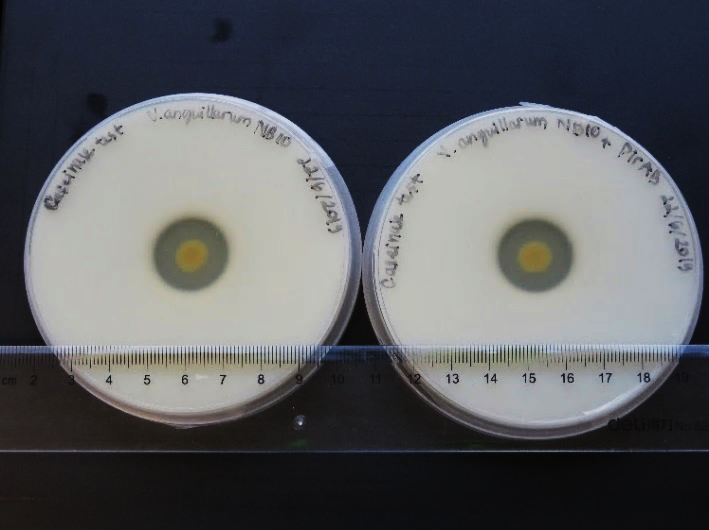** | | **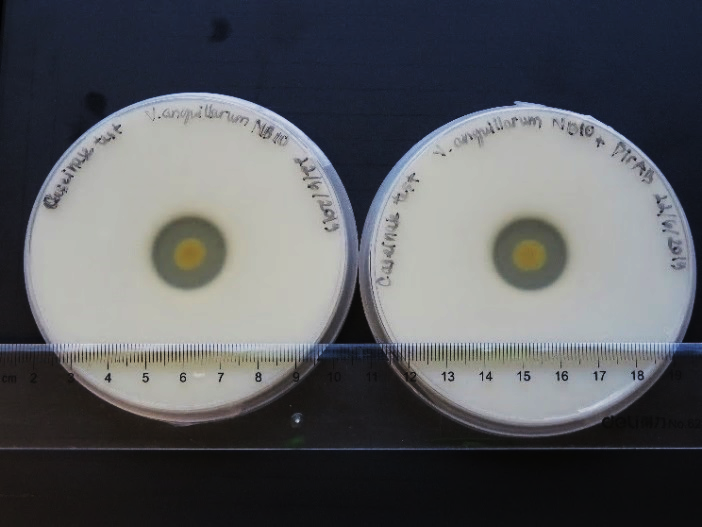** | | |  | |
| Lipase | | **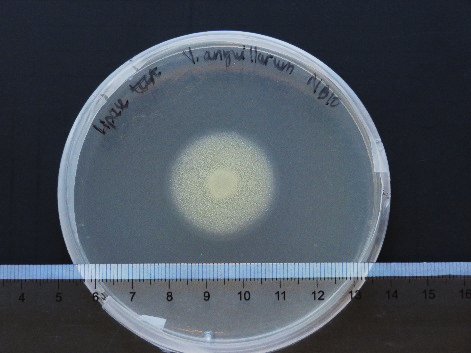** | | **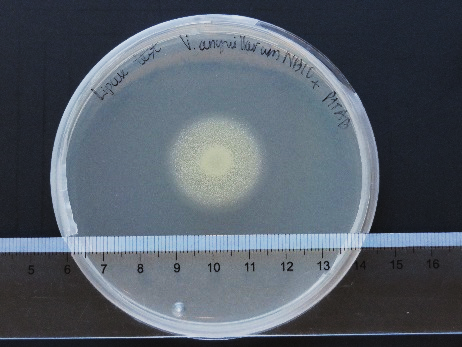** | | | No activity (No opalescent zone around the colony) | |
